# WSN–IoT-Based Mobile Environmental Monitoring System for Smart Cities

**DOI:** 10.3390/s26216701

**Published:** 2026-10-23

**Authors:** Erick Martínez, Maira Gasca, Luis Camargo

**Affiliations:** 1 Faculty of Engineering, Universidad del Magdalena, Santa Marta 470004, Colombia; erickmartinezd@unimagdalena.edu.co (E.M.); mgasca@unimagdalena.edu.co (M.G.); 2 School of Basic Sciences, Technology and Engineering, Universidad Nacional Abierta y a Distancia, Bogotá 111511, Colombia

**Keywords:** mobile wireless sensor networks, Edge–Fog–Cloud, geospatial monitoring, iterative and incremental development, spatial segmentation, MQTT over WebSockets, metrological validation

## Abstract

In smart cities, reliable environmental information is essential for decision-making. Environmental monitoring using fixed stations presents limitations in spatial coverage. This work presents an environmental monitoring system for precipitation, PM_2.5_, PM_10_, CO_2_, global irradiance, temperature, barometric pressure, and wind speed, based on an Edge, Fog, and Cloud architecture. The system consists of a mobile sensor node that integrates the sensors and performs spatially resolved measurements, and a fixed sink node that forwards the data to an IoT broker and a database. In addition, a web application was developed for MQTT message visualization, geospatial queries, and measurement download. The nodes were developed using an iterative and incremental methodology based on agile principles, pragmatic programming, and a Bottom-Up strategy. The nodes incorporate spatial segmentation, intermediate storage, automatic retransmission, integrity verification, and flow control mechanisms. Laboratory and real-world tests confirmed TRL 6. Metrological performance evaluation using independent validation showed reduced RMSE for four of six variables, with the greatest improvement for PM_2.5_. CO_2_ measurements had a coefficient of variation below 1.3%, and the rain gauge a previously determined relative error of 8.5%. These results demonstrate the feasibility of the system for extending the spatial coverage of urban environmental monitoring through mobile sensing.

## 1. Introduction

Several authors agree that the concept of smart and sustainable cities constitutes a governance paradigm aimed at mitigating the problems arising from urban population growth and accelerated urbanization processes [1,2]. This management approach considers the technological factor as a cross-cutting axis of the city’s other components, insofar as it enables the monitoring and interconnection of its systems, connects citizens with their surroundings, and automates specific processes [3,4]. In this context, Information Technology (IT) enables the collection, transmission, processing, and analysis of data to support decision-making and resource management in a transparent and efficient manner, with the aim of planning a city that improves the quality of life of its inhabitants and ensures sustainable development [4,5,6].

The use of technology for monitoring environmental variables in a city can impact its collective intelligence, particularly in the categories of Environment, Health, Governance, and Urban Planning [6]. The data collected through monitoring are transformed into information and subsequently into policies and improvement actions within these categories [5].

One of the main challenges is to design and implement an efficient urban environmental monitoring system with minimal technological infrastructure requirements for the city.

One solution for monitoring variables is the implementation of a Wireless Sensor Network (WSN) [7]. WSNs consist of sensor nodes that incorporate a wide range of measurement devices, computing and communication capabilities, and sink nodes or cluster heads that collect the data transmitted by the sensor nodes and relay them to the monitoring center. The sensor nodes are deployed throughout the city, ensuring measurement coverage and connectivity while facing the challenge of using the smallest possible number of nodes [7,8]. In contrast, sink nodes in static and hierarchical WSNs are strategically deployed to organize the network into clusters, where each cluster head manages a defined group of sensor nodes. This arrangement optimizes energy efficiency by minimizing multi-hop communications, thereby reducing battery consumption, improving link stability, and decreasing latency. Recent approaches, such as those based on artificial intelligence and quantum-inspired computing algorithms, confirm that cluster management is essential for maximizing the lifetime of the WSN [9,10].

The introduction of mobility into WSNs (MWSNs) enables the expansion of network coverage and enhances the scope of applications. It also mitigates congestion and improves energy efficiency [11]. MWSNs are versatile in both topology and implementation. Mobility may be provided by the sink node to efficiently collect data from fixed sensor nodes [12]; by mobile sensor nodes and fixed sink nodes to provide greater measurement coverage of the monitored variables [13]; or by mobile nodes with sensing and relaying capabilities that do not rely on fixed infrastructure, dynamically forming an ad hoc network [14].

WSNs implemented as Mobile Ad hoc Networks (MANETs) present significant challenges due to the absence of fixed infrastructure, a base station, or an access point. These challenges are particularly evident in the routing protocol, since communication is performed through multiple hops and aspects such as energy efficiency, the distance between nodes, topology variability, and node speed must be considered [15,16]. In addition, the implementation of these protocols requires greater processing capability in the nodes than in a hierarchical WSN, because they must simultaneously operate as sensing nodes and routers within a flat architecture with no defined hierarchy [17]. This type of network is common in scenarios where deploying fixed infrastructure is not feasible, such as emergency operations, military environments, or remote areas [18]. However, given the characteristics of urban environments and the requirements of the proposed system, this architecture was not considered in the design.

In WSNs with mobile sink nodes, Unmanned Aerial Vehicles (UAVs) are widely used. They collect data from large areas, avoid ground obstacles, establish line-of-sight communication with ground nodes, and reduce the energy consumption of fixed sensor nodes by decreasing the number of hops and retransmissions. Several studies have reported their applicability in smart cities, while others focus on optimizing the number of required UAVs, the UAV flight path for data collection, and the routing protocol [19,20,21,22]. This architecture was not considered in the proposed system because it is intended for scenarios in which the area is already covered by sensor nodes and the objective is to collect the data efficiently. In the specific case addressed in this work, one of the main limitations is the limited number of sensor nodes available to cover the urban area.

WSNs with mobile sensor nodes offer significant advantages over traditional fixed monitoring stations by providing greater spatial coverage with less deployed infrastructure [23,24,25]. This topology is particularly relevant in the context of smart cities, where sensor mobility increases the effective spatial resolution and the spatial representativeness of the collected data [23].

Figure 1 shows the topologies corresponding to the different options considered for implementing a WSN.

The proposed system consists of a WSN with mobile sensor nodes equipped with different low-cost sensors (LCSs). These LCSs measure precipitation, PM_2.5_, PM_10_, CO_2_, solar radiation, temperature, atmospheric pressure, and wind. In addition, they are installed on a platform mounted on buses of the public transportation system, with the aim of providing measurement coverage throughout the study city using a limited number of nodes. This strategy is based on the fact that public transportation repeatedly travels through strategic locations across the city at different times of the day, making it possible to perform measurements while in motion and obtain a more dynamic characterization of the monitored environment. Likewise, static sink nodes are deployed at road intersections with high public transportation traffic.

As in traditional WSNs, in an MWSN with mobile sensor nodes, duty cycle, latency, and packet loss are interrelated and introduce challenges in system design. On the one hand, reducing the duty cycle decreases energy consumption by keeping the nodes in a sleep state for longer periods [26]; however, a mobile node in a sleep state may pass through the coverage area of a sink node without having an effective opportunity to communicate. On the other hand, movement may cause link interruptions during transmission and the consequent packet loss [23]. Whether due to the sleep state or the absence of coverage, when data cannot be transmitted in a timely manner, latency increases and local storage is required until communication is restored. Although this storage mitigates information loss, insufficient memory capacity may cause buffer overflow and the consequent loss of data [27]. To address these challenges, it was determined that the Mobile Sensor Node should remain continuously active throughout its operation, ensuring its autonomy through a photovoltaic system with battery storage. In the event of connectivity interruptions, intermediate storage was implemented in both the sensor node and the sink node, complemented by data transport reliability mechanisms to prevent information loss. As a trade-off of this design decision, variable delivery latency is accepted, since frames generated outside the coverage area must remain stored until the mobile node re-establishes communication with a sink node.

Mobile monitoring of environmental variables has been used in various urban environments to complement stationary measurements, particularly for air pollution assessment using LCSs [28]. These moving LCSs make it possible to capture hyperlocal, street-scale variations in pollutants [29]. Although pollutants such as PM_2.5_ exhibit greater variability at the regional scale, others associated with traffic, such as NO_2_ and CO, may experience significant fluctuations over distances of only a few meters [30,31]. These spatial dynamics can be analyzed to support decision-making in critical areas and time periods in terms of mobility, pollution, and public health.

In mobile monitoring, vehicle movement introduces challenges in data consistency and adjustment, as well as in sensor calibration. This sensing modality faces technical issues such as measurement variability induced by vehicle speed, sensor orientation with respect to airflow, mechanical vibrations, and abrupt changes in the microenvironment [28,29]. Likewise, in dense urban environments, interruptions in wireless communication and GPS georeferencing errors compromise measurement accuracy. In addition, the spatial resolution of the measurements depends on vehicle speed and the response times of the sensors; an increase in speed may result in undersampling, preventing the acquisition of measurement records for a specific georeferenced area.

Since few LCSs have been designed to operate while in motion, these limitations highlight the need to adapt calibration models and data adjustment techniques that account for mobility-induced variability and sensor asynchrony.

Several calibration strategies can be implemented in mobile environments. One of the most widely used is direct field calibration, which is based on the co-location of the sensor (mobile platform) with a fixed reference station to obtain reliable calibration functions [32]. However, this strategy may become costly when a large number of sensors must be calibrated. As an alternative, transfer-based calibration and proxy-based calibration can be employed. Both approaches rely on the prior establishment of a calibration function through direct field calibration. In the proxy-based approach, the previously calibrated node is co-located with uncalibrated sensors, allowing the calibration function to be transferred indirectly. In contrast, transfer-based calibration does not require co-location, since the calibration function is applied directly to sensors of the same type. For the proper implementation of these approaches, similar climatic conditions and comparable pollutant emission sources are required in the environments where the sensors will operate [33,34]. In addition, their performance may be affected by the propagation of cumulative errors [29].

To improve the stability of the measurements, eliminate errors and false peaks induced by mobility and environmental variability, and provide data consistency and smoothing, traditional techniques such as the simple moving average, which averages the measurements to reduce fluctuations; the exponential moving average, which assigns greater weight to recent measurements to capture changes in microenvironments; the median filter, which is effective in removing inconsistent peaks; and the Kalman filter, which provides immediate adjustment and dynamic smoothing without relying on the prior accumulation of data, can be employed. Likewise, modern machine learning-based techniques, such as random forests, recurrent neural networks, and support vector machines, can also be integrated to improve accuracy by modeling complex and nonlinear patterns in the measurements [35,36,37].

Regarding the calibration (accuracy adjustment) of the proposed system prototype, the mobile sensor node was subjected to two complementary calibration schemes. Field calibration was performed through direct comparison with a fixed reference using instrumentation under stationary conditions. Simple linear regression was performed, and this operation was executed in the processing unit of the sensor node. This made it possible to adjust the sensor sensitivity using the slope and correct the baseline offset using the intercept. Furthermore, whenever physical or electronic modifications were made to the sensors, recalibration procedures were carried out in the laboratory under controlled conditions, ensuring the internal traceability of the system before its field deployment. Calibration function transfer was not performed because only one node was implemented. However, direct transfer calibration was planned for future nodes, provided that the same electronic devices are used.

To optimize accuracy, machine learning techniques and advanced filters, such as the Kalman filter, were not implemented at this stage of the project for data smoothing and adjustment. Instead, simple and transparent processing methods, such as averaging and the median filter within each spatial resolution cell, were employed, particularly when differences existed in the sensor response times, that is, when one sensor generated many data points while another generated only a few or a single data point.

This decision was based on two technical criteria: preserving the dynamic behavior of the system during the experimental phase and avoiding the computational overhead on the mobile node, while prioritizing energy efficiency and operational stability. The characterization of the city’s environments and the prediction of environmental variables will be carried out as future work using artificial intelligence. Their implementation is planned for the deployment phase of the monitoring center, where greater computational capacity will be available, rather than in the sensor node or the sink node.

The Edge/Fog/Cloud architecture provides a flexible and scalable framework that meets the requirements of WSN-based IoT systems [38]. This functional separation preserves the autonomy of Edge devices and eliminates critical transmission bottlenecks through the integration of broadband networks and protocols such as MQTT at the Fog layer [39,40]. In addition, by bringing computation closer to the data source, system efficiency is improved [41]. Finally, the Cloud is reserved for high-demand tasks such as large-scale data storage and the execution of artificial intelligence algorithms [38,39,42].

The proposed system implements this hierarchical architecture: the mobile sensor nodes operate at the Edge, dedicated exclusively to data acquisition and transmission; the sink node acts as the Fog layer, managing data aggregation and the secure transmission of information; and finally, the Cloud infrastructure supports historical data management and advanced processing for the monitoring center.

The Internet of Things (IoT) extends connectivity to the physical environment with minimal human intervention, enabling objects and processes to interact through publish/subscribe models [43,44]. Unlike conventional technologies, IoT enables interoperability among heterogeneous devices and supports distributed management [45], transforming data flows into scalable, event-driven services.

The proposed system employs IoT and the MQTT protocol over an open-source broker to optimize messaging, enabling notifications of critical changes in a specific variable or at specific locations according to the user’s subscription. This architecture is integrated with a NoSQL database for large-scale data storage and a protocol adapter that ensures interoperability with HTTP-based client applications. In this way, environmental data can be used by actuators or visualized by users, such as citizens and municipal authorities, through IoT publish/subscribe protocols.

Considering the above, the objective of this work is to describe the development and operation of a mobile environmental monitoring system based on WSN and IoT in the context of a city with limited technological infrastructure. Under this approach, the present study reports several key findings, which are summarized as follows:

(1) A mobile WSN–IoT architecture that enables environmental monitoring across the entire city using a smaller number of sensors through mobility.
(2) Data correction and synchronization methods that ensure reliable, georeferenced, and time-stamped measurements under mobile sensing conditions.
(3) A methodological approach for the development of prototypes incorporating both hardware and software.
(4) Experimental validation using a mobile platform, achieving a Technology Readiness Level (TRL) of 6 with low measurement error compared with fixed sensors.

The following sections present a brief literature review, the methodological framework, and the design adopted for the development of the prototypes. Subsequently, the results obtained from the implementation of the system are presented, together with the corresponding discussion and future work aimed at improving the proposed system.

## 2. Related Work

Recent literature on WSN- and IoT-based environmental monitoring shows growing interest in mobile architectures, calibration strategies, and distributed processing. To provide context for the proposed system and identify the strategies and architectures employed in this field, a literature review was conducted focusing on static and mobile WSNs, calibration mechanisms, Edge/Fog/Cloud architectures, and mobile environmental sensing prototypes.

The selected studies were organized and analyzed according to their technical contributions, making it possible to identify strategies, methodologies, and technologies that could be incorporated, adapted, or discarded within the proposed architecture.

Initially, studies aimed at ensuring sensing coverage in static WSNs were reviewed. Reference [7] presents a review of algorithms for optimizing sensor node placement, whereas [8] proposes an energy-efficient and reliable coverage method based on an improved Grey Wolf Optimization algorithm to ensure that each monitoring point is covered by at least one sensor node. In addition, refs. [9,10] investigate cluster-head selection strategies and clustering algorithms for balancing energy consumption in hierarchical WSNs using artificial intelligence and quantum-inspired algorithms, respectively. Collectively, these studies demonstrate that coverage optimization and clustering remain critical components for maximizing the energy efficiency and lifetime of WSNs. In the context of the proposed system, these approaches could be applied in future stages to determine the optimal locations of sink nodes at the city’s road intersections.

Subsequently, studies related to the incorporation of mobility into WSNs were analyzed. Reference [11] presents a review of mobility algorithms applicable to sink nodes, sensor nodes, and repair or reconnection nodes. Since WSNs constitute one of the technological foundations of IoT, many of these strategies could be adapted to modern IoT architectures.

Within this context, proposals specifically focused on mobile sink nodes were analyzed. In [12], route optimization algorithms are classified according to static, dynamic, and hybrid trajectories, as well as according to selection criteria based on events, path constraints, time periods, and routing techniques. Likewise, refs. [20,21,22] explore the use of UAVs as mobile sink nodes. These studies analyze variables such as trajectory, altitude, speed, transmission power, and energy consumption, as well as air-to-ground and ground-to-ground communication protocols aimed at minimizing energy consumption through dynamic cluster reorganization and adaptive cluster-head selection. However, most of these proposals assume dense sensor deployments and focus primarily on optimizing data collection. These findings could be useful in future scenarios in which mobile sensor nodes and mobile sink nodes operate together with higher-capacity fixed sink nodes interconnected through broadband technologies, such as cellular networks, point-to-point wireless links, or optical fiber. In addition, ref. [41] proposes a sink node approach algorithm and a routing mechanism based on hop count and energy consumption, introducing a multi-input, multi-output architecture for cloud connectivity.

Studies on dynamically ad hoc sensor networks implemented under the MANET paradigm were also examined. In [14,15,16,17,18], routing protocols for mobile nodes with sensing and relay capabilities are presented. References [15,16] correspond to literature reviews, whereas [14] proposes a protocol for underwater communications using acoustic signals instead of radio frequency. In turn, ref. [17] proposes an energy-efficient heterogeneous clustering approach, and [18] presents a priority-aware protocol for emergency scenarios. However, these proposals were excluded because they are not aligned with the architecture proposed for a smart city, which is based on fixed infrastructure to facilitate data aggregation, synchronization, and management.

More specifically, studies related to mobile sensor nodes, considered the initial solution of the proposed system, were reviewed. In [13], a method based on Compressive Sensing is proposed to construct scalar maps from data collected by mobile sensor nodes at unknown locations. In [23], a model is developed to calculate the probability of successful communication by considering the sensor node speed and payload size in the presence of static sink nodes. Likewise, ref. [25] investigates environmental pollution monitoring using Vehicular Sensor Networks (VSNs), whereas [29] reviews the evolution of low-cost sensor networks for air quality monitoring, emphasizing the challenges associated with mobile monitoring. Collectively, these studies demonstrate that sensor node mobility improves the spatial representativeness of the measurements but introduces challenges related to connectivity, synchronization, calibration, and the consistency of the collected data.

Due to the particular characteristics of measurements acquired in motion, studies focused on calibration, data processing, and data completion were also analyzed. In [29], the challenges associated with calibrating mobile sensors operating at different speeds are discussed, and data smoothing techniques are presented. In [30], a scalable geospatial framework is proposed to improve the reliability of PM_2.5_ data obtained through Crowdsourcing using low-cost sensors, introducing concepts of spatial correlation and spatial similarity based on Euclidean distance. In [32], the Multi-Hop Calibration technique is validated for mobile sensors, demonstrating its ability to reduce cross-sensitivities and meteorological dependence. In addition, ref. [34,37] compare calibration models based on multivariate regression and machine learning, identifying the Random Forest algorithm as the best-performing approach for low-cost PM_2.5_ sensors. Finally, ref. [36] proposes a missing data imputation strategy based on moving averages and linear interpolation to compensate for information loss caused by vehicle speed, sensor temporal resolution, or prototype electronic failures. Collectively, these studies demonstrate that calibration and data processing remain critical components of mobile monitoring systems based on low-cost sensors, particularly under dynamic operating conditions. Furthermore, these studies show that these solutions can be implemented either locally on the sensor node or in higher layers within a hierarchical architecture.

Finally, studies related to data acquisition, transmission, and management in WSNs integrated with IoT were reviewed, revealing a trend toward hierarchical Edge/Fog/Cloud architectures. In [38], an architecture of this type is successfully evaluated for monitoring environmental variables in buildings using low-cost devices at the Edge layer and single-board computers, such as Raspberry Pi, at the Fog layer. Similarly, ref. [40] implements an Edge/Fog/Cloud-based IoT architecture incorporating MQTT at the Fog layer using Raspberry Pi, a technological profile similar to that adopted in this work. Likewise, ref. [39] reviews WSN–IoT integration, highlighting the need to incorporate high-data-rate wireless technologies, such as WiFi, 5G, D2D, and SDN-based networks for WSNs, to reduce latency. Collectively, these studies demonstrate that the Edge/Fog/Cloud architecture adopted in the proposed system facilitates the integration of WSNs and IoT by distributing functions across different layers and enabling the incorporation of different bearer services according to the connectivity requirements of each layer.

Complementarily, articles related to prototypes developed for measurements acquired in motion were reviewed. In [24], mobile environmental monitoring systems based on sensors installed on vehicles and communication technologies such as ZigBee and GSM are presented. Likewise, ref. [28] investigates the portability of PM, NO_2_, and BC sensors for mobile urban monitoring applications. In addition, ref. [46] describes the development of a precipitation sensor designed to operate under mobile conditions. In [47], although no prototype is developed, the Aclima mobile laboratory measurement and acquisition platform, installed on vehicles, is used to evaluate measurement performance through comparison with fixed reference stations. The study that is most similar to the proposed system is [48], which presents an urban sensing system based on sensors installed on public service vehicles and an Edge/Fog/Cloud architecture, using LoRaWAN for communication between the mobile nodes and the Fog infrastructure, together with temporary storage mechanisms during coverage interruptions. Collectively, these studies demonstrate the feasibility of mobile environmental monitoring and show different strategies for the acquisition, transmission, processing, and evaluation of measurements.

Table 1 presents a comparison of similar studies that implement environmental monitoring systems on mobile platforms.

Unlike the reviewed studies, the present work does not focus exclusively on a particular system component, but rather on the vertical and functional integration of multiple technologies and methodologies for multivariable mobile environmental monitoring. The contribution does not lie in the individual technologies employed, but in their integration within an MWSN–IoT Edge–Fog–Cloud architecture, developed using an iterative and incremental methodology for the development of the hardware and software prototypes and their integration with Cloud services and the client application. The system implements mechanisms to ensure its operation during mobility and prevent data loss, and its experimental validation in a real urban environment enabled the achievement of Technology Readiness Level (TRL) 6.

## 3. Materials and Methods

The following section describes the methodological design adopted in this study and the behavioral description of the proposed system.

### 3.1. Methodological Design

Prototypes are used to evaluate the performance of a technology before its production and commercialization. For a development to be considered a viable prototype, it must successfully undergo feasibility testing under operating conditions within a relevant environment, achieving at least Technology Readiness Level (TRL) 6 [49].

The prototypes developed for the mobile sensor node and the sink node involve the simultaneous integration of hardware and software. The node hardware consists primarily of electronic components, together with electrical and mechanical elements. At least one electronic component is capable of, and required to, execute a computational algorithm to operate correctly within the system; this software is implemented as firmware. In addition, communication technologies are incorporated for the interconnection of chips and peripherals, such as I2C, UART, and USB; Ethernet modules for integration with the network infrastructure; and wireless communications for node-to-node interaction, such as XBee for low-power operation and WiFi for higher data transmission rates as a wireless alternative to Ethernet. In turn, the monitoring center is a software prototype that implements different types of services on different platforms. Likewise, the system incorporates distributed processing across the sensor node, the sink node, and the monitoring center, as well as validation under dynamic operating conditions. These characteristics introduce complexities that are not fully addressed by traditional sequential development methodologies, particularly because of the need to progressively integrate heterogeneous subsystems and perform iterative testing related to synchronization between physical and logical components, data acquisition, calibration, mobility, data processing and measurement correction, georeferencing, and communication between devices.

In addition, the system behavior depends on real-world variables, such as travel speed, wireless coverage, vibration, and latency, which require incremental validation and continuous feedback throughout the development process. For this reason, an iterative and incremental methodology was designed to reduce integration risks and experimentally validate each subsystem before its incorporation into the complete system.

The proposed methodology is based on agile development principles, pragmatic programming, incremental hardware development using the Bottom-Up strategy, and progressive hardware–software integration techniques. From the agile approach, concepts such as iterative development, flexibility to accommodate changes during system development, collaborative work, and prioritizing functional solutions over rigid processes and extensive documentation are adopted [50,51]. From pragmatic programming, strategies aimed at early fault detection and correction, progressive refactoring, and functional validation through preliminary system versions are incorporated. Likewise, principles such as iterative experimental learning and the use of Tracer Bullets are employed to enable early validation of the system’s functional architecture [52]. Furthermore, the Bottom-Up strategy allows basic components to be developed first and subsequently integrated into more complex subsystems, facilitating synchronization among firmware, electronics, and communications [53,54]. Finally, functional validation mechanisms and testing interfaces are incorporated to monitor the system behavior throughout the different stages of integration and verification [55].

The methodology is shown in Figure 2 and is organized into three main phases: initialization, development, and verification.

The initialization phase comprises the storytelling, analysis, and Tracer Bullet stages. During this phase, the facts describing the problem and the potential solutions to be provided by the prototype are identified. Likewise, the functional and non-functional requirements of the system, together with the development objectives, are established. Finally, the behavioral and structural descriptions of the prototype are developed by decomposing it into different components, and the corresponding tasks and roles are assigned within the development team. This phase concludes with a Tracer Bullet, which consists of a simplified version of the hardware or software that guides the development process and enables early validation of the proposed architecture.

The development phase follows an iterative and incremental approach and comprises multiple sprints. Each cycle represents the development and integration of an element, subsystem, or system into the final product. These sprints may be carried out in parallel or sequentially; however, the complexity of the product increases with each iteration. This phase begins with the development of elements, followed by subsystems, then systems, and so on until the final prototype is achieved. Each iteration includes construction, integration, and laboratory testing activities. The construction stage is also carried out through iterations or sprints, which may involve the exclusive development of hardware, software, or both simultaneously. When a component requires both hardware and software, these developments must be performed in parallel and synchronized upon completion of one of the defined cycles. For example, a component may require two software sprints and one hardware sprint, or vice versa. In any case, integration is performed only when both developments reach a compatible functional level. In addition, each cycle of this stage has its own rules, procedures, and constraints associated with the component being developed.

During the integration stage, the components are required to comply with the specifications defined in the Tracer Bullet, such as data format, communication bus, ports, power supply levels, and the number of terminals or physical connections, among others. Subsequently, during the laboratory testing stage, the integrated operation of the system is validated. For this purpose, a graphical user interface is used to monitor the data, and measurement instruments are used to verify the behavior of the signals and electrical variables involved.

The verification phase begins once the complete prototype has been successfully validated in the laboratory during the previous phase and ends when it is successfully evaluated under real operating conditions. If the demonstration results are not satisfactory, the development phase or the initialization phase must be revisited, depending on the severity and complexity of the identified problem.

The verification phase comprises the demonstration and delivery stages. During the demonstration stage, the system is installed and operated for a sufficient period of time to expose it to the different operating and environmental conditions expected during its intended use. The purpose of this stage is to validate the prototype performance in a relevant operational environment and verify compliance with the established functional and non-functional requirements. The results obtained provide the experimental evidence necessary to demonstrate Technology Readiness Level (TRL) 6. Subsequently, the executables, source code, drawings, component lists, system diagrams or representations, test result records, and the system manual are documented and delivered.

This methodological design proved particularly useful during the development of the proposed system because of the need to integrate environmental sensors, mobile platforms, wireless communications, and IoT services within a distributed architecture.

#### Application of the Proposed Methodology

The development procedure applied the proposed methodology following the three phases described in the previous section.

- Initialization Phase

During the initialization phase, the Tracer Bullet was defined through the Storytelling and Analysis stages, establishing the WSN topology with mobile sensor nodes and static sink nodes, as shown in Figure 1c, the Edge/Fog/Cloud architecture, the main communication protocols among its components, and the environmental variables to be incorporated into the monitoring system. This constituted the baseline for the subsequent development and integration cycles.

- Development Phase

Subsequently, during the development phase, the multiple sprints were grouped according to the architecture defined by the Tracer Bullet. Initially, the sprints were focused on the development of the Mobile Sensor Node (Edge), progressively incorporating the environmental sensors and their corresponding data acquisition routines into the firmware. Once the sensors had been integrated, functional validation was performed under laboratory conditions.

Figure 3 illustrates the evolution of the Mobile Sensor Node throughout this process.

During the same development phase, the subsequent sprints were dedicated to the Sink Node (Fog). Initially, the radio-frequency communication subsystem was integrated with the processing unit, and subsequently, the functions for receiving, validating, temporarily storing, and retransmitting the frames received from the mobile node were implemented in the firmware. Subsequent iterations enabled the integration of both nodes, verification of their interoperability, refinement of the communication protocols, and adaptation of the measurement format for publication to and storage in the Cloud infrastructure.

Finally, the last sprints of the development phase were dedicated to the implementation of the Monitoring Center (Cloud), progressively incorporating the IoT service, the database, the application server, and the web application. In each iteration, the correct operation of the publication, storage, retrieval, and visualization of the environmental measurements was verified, as well as the integration of all the software components of the system.

- Verification Phase

During the verification phase, functional validation was carried out by operating the complete system, initially under laboratory conditions, where measurements were taken, the correct visualization of the interfaces was verified, and data traceability and integrity were assessed by reviewing the temporary files generated by each component (measurement records, frames at the Mobile Sensor Node and the Sink Node, and published and stored values). Subsequently, test runs were conducted using the vehicle under real operating conditions. These tests made it possible to validate the interaction among the Edge, Fog, and Cloud layers, as well as the structural integrity of the Mobile Sensor Node.

Once the prototype had been functionally validated, a calibration campaign was conducted to improve the accuracy of the measurements before releasing the final version of the prototype. The resulting calibration models were subsequently incorporated into the firmware, which required an additional development iteration before the release of the final prototype.

The calibration functions are linear regressions obtained by comparing the measurements acquired by the Mobile Sensor Node with the reference values provided by the UNIMAG KOICA air quality monitoring station, located at the experimental agricultural farm on the campus of the Universidad del Magdalena in the city of Santa Marta. The station is owned by the Colombian Institute of Hydrology, Meteorology and Environmental Studies (IDEAM), and its data are available through the Air Quality Information Subsystem (SISAIRE).

To ensure that both systems were exposed to the same environmental conditions, the Mobile Sensor Node remained stationary next to the reference station during the data acquisition process for calibration, as shown in Figure 4.

During the calibration process, the Mobile Sensor Node generated one data frame every five minutes, whereas SISAIRE publishes a record of the environmental variables with a temporal resolution of one hour. To make both datasets comparable, the data frames generated by the mobile node were preprocessed by grouping them into hourly intervals and calculating the arithmetic mean of the measurements for each variable. Subsequently, the variables reported by SISAIRE were converted to the same units used by the Mobile Sensor Node before performing the regression analysis.

During the calibration process, the firmware of the Mobile Sensor Node was temporarily modified to generate one data frame every five minutes, whereas SISAIRE publishes a record of the environmental variables with a temporal resolution of one hour. To make both datasets comparable, the data frames generated by the mobile node were preprocessed subsequently during data analysis by grouping them into hourly intervals and calculating the arithmetic mean of the measurements for each variable. The calibration campaign was conducted between 7:00 a.m. and 7:00 p.m., yielding twelve consolidated hourly records paired with the reference station records, with the first consolidated record obtained at 8:00 a.m. Subsequently, the variables reported by SISAIRE were converted to the same units used by the Mobile Sensor Node before performing the linear regression analysis. The paired records were used to obtain the calibration model parameters (slope, intercept, and R^2^). In addition, RMSEraw and MNBraw were calculated for this dataset before applying the calibration, whereas RMSEcal, MNBcal, and NRMSE were calculated after applying the calibration functions. This procedure was performed for the variables for which reference measurements were available: temperature, barometric pressure, wind speed, global irradiance, PM_2.5_, and PM_10_. To independently validate the models, a second measurement campaign was conducted on a different day, following the same data acquisition and consolidation procedure. This second campaign yielded nine independent paired hourly records, corresponding to the consolidated records from 8:00 a.m. to 4:00 p.m. These records were not used to refit the calibration models but exclusively to evaluate their performance using RMSE, NRMSE, and MNB.

(1) $${RMSE}_{raw}=\sqrt{\frac{1}{n}\sum_{i=1}^{n}{\left(x_{i}-y_{i}\right)}^{2}}$$

(2) $${RMSE}_{cal}=\sqrt{\frac{1}{n}\sum_{i=1}^{n}{\left({\hat{y}}_{i}-y_{i}\right)}^{2}}$$

(3) $${MNB}_{raw}\left(\%\right)=\frac{100}{n}\sum_{i=1}^{n}\frac{x_{i}-y_{i}}{y_{i}}$$

(4) $${MNB}_{cal}\left(\%\right)=\frac{100}{n}\sum_{i=1}^{n}\frac{{\hat{y}}_{i}-y_{i}}{y_{i}}$$

(5) $${NRMSE}_{j}=\frac{{{RMSE}_{j}}_{cal}}{y_{{max}_{j}}-y_{{min}_{j}}}$$

where n is the number of measurements of the environmental variable being evaluated, $y_{i}$ is the reference measurement obtained from UNIMAG KOICA, $x_{i}$ is the sensor measurement before applying the calibration equation, and $\hat{y_{i}}$ is the value estimated by the calibration model.

### 3.2. System Components

This section describes the main components of the proposed system and their functionalities.

#### 3.2.1. Mobile Sensor Node Components (Edge Device)

The Mobile Sensor Node consists of the processing, power supply, RF communications, geolocation, data acquisition, user interface, and environmental sensing subsystems. These subsystems were designed using a modular and integrated approach, resulting in a scalable architecture that facilitates the incorporation, replacement, or upgrading of components without modifying the overall operation of the node. Additionally, the node includes a plastic enclosure to provide mechanical protection for some components and an air extractor to maintain the internal temperature and prevent overheating.

The processing subsystem was implemented using a Raspberry Pi 4 Model B single-board computer equipped with a 1.5 GHz quad-core processor and 4 GB of RAM. This serves as the central unit of the node, integrating data acquisition, local storage, and local data processing. In addition, it manages the I^2^C, USB, and GPIO interfaces for connecting sensors and peripherals, the RF communications module, and the user interface.

The power supply subsystem consists of a 20 Wp, 12 Vdc polycrystalline solar panel, a charge controller, and a 12 Vdc sealed battery. The solar panel serves as the primary energy source for the sensor node, while the charge controller manages the charging process and protects the battery during operation. The battery provides the energy storage required to ensure the continuous operation of the system in the absence of solar radiation or during temporary variations in photovoltaic power generation. Additionally, this subsystem provides energy autonomy to the sensor node, eliminating dependence on the vehicle’s electrical system during operation.

The RF communications subsystem was implemented using a Digi XBee PRO S3B module (Digi International Inc., Hopkins, MN, USA) operating in the 902–928 MHz ISM band, with a maximum transmission power of 24 dBm and a 2.1 dBi ANRP900/2.1 external antenna. This subsystem provides the wireless link between the Mobile Sensor Node and the Sink Node when the vehicle passes through an intersection where a Sink Node is installed, enabling the transmission of the data acquired and stored during the previous route. This strategy allows data transmission to occur only within the coverage area of the Sink Node, eliminating the need for a continuous citywide communications infrastructure and reducing the energy consumption of the sensor node. Communication between the Raspberry Pi and the XBee module is established through a USB-to-UART interface.

The geolocation subsystem was implemented using a u-blox NEO-M8M GNSS receiver (u-blox AG, Thalwil, Switzerland) connected to the Raspberry Pi through a USB-to-UART interface. The receiver supports multiple global navigation satellite system (GNSS) constellations, including GPS, GLONASS, Galileo, BeiDou, and QZSS. It provides 72 channels, a typical horizontal accuracy of 2.5 m CEP (Circular Error Probable), and a maximum update rate of 10 Hz. This subsystem provides the geographical position and time reference for each measurement. Additionally, it provides the vehicle speed, which is used during the processing and correction of measurements associated with platform mobility.

The data acquisition subsystem was implemented using 16-bit ADS1115 analog-to-digital converters (Texas Instruments Incorporated, Dallas, TX, USA) and USB-to-UART converters. The ADS1115 converters enable the acquisition of signals from sensors with analog outputs, providing four general-purpose inputs or two differential inputs, a maximum sampling rate of 860 SPS, and a programmable gain amplifier (PGA). In turn, the USB-to-UART converters enable the integration of sensors and devices with serial communication into the Raspberry Pi USB bus. The use of USB interfaces made it possible to incorporate an externally powered USB hub, increasing the number of connected devices, relieving congestion on the Raspberry Pi native ports, and preventing overloads in its power supply caused by powering multiple peripherals. Overall, this subsystem provides a unified interface for acquiring data from sensors with different output technologies, facilitating their integration and processing within the sensor node. As a result, all sensors in the node are integrated through standardized I^2^C or USB communication buses, simplifying the hardware architecture, facilitating the incorporation or replacement of sensors, and improving the scalability of the system.

The user interface subsystem was implemented using a 7-inch capacitive touchscreen connected to the Raspberry Pi through the HDMI and USB interfaces, together with a wireless keyboard equipped with a USB receiver. This subsystem provides a local interface for configuring, operating, and maintaining the sensor node, allowing users to monitor the system status, verify data acquisition in real time, and configure the communication parameters of the XBee module. Additionally, during prototype development, this interface was used for firmware debugging, validation of new functionalities, and execution of field tests, enabling adjustments to be made directly on the sensor node without returning to the laboratory. This capability facilitated the iterative development process, significantly reduced system integration and validation time, and accelerated prototype development.

The environmental sensing subsystem consists of the sensors responsible for measuring the environmental variables. The sensors used and their main characteristics are presented in Table 2.

The Mobile Sensor Node firmware was developed in Python 3.8 running on Raspberry Pi OS and controls the configuration, acquisition, processing, storage, and transmission of environmental measurements.

Initially, the firmware configures the Digi XBee PRO S3B communications module by setting parameters such as the network identifier (ID) to which the Sink Node and the Mobile Sensor Node belong, as well as the high and low address fields (DH and DL) that identify the Sink Node XBee module. Subsequently, the firmware verifies the connection and proper operation of all sensors, preventing the data acquisition process from starting until every device is connected and operational, thereby avoiding incomplete data frames.

During operation, the firmware executes independent routines for each sensor to obtain the corresponding environmental variable. These routines are adapted to the functional characteristics and measurement principle of each device. Depending on the sensor, the firmware configures the analog-to-digital converter (ADC), establishes communication through the corresponding protocols, or executes the command sequences required to initiate data acquisition. Subsequently, the acquired information is processed according to the corresponding instrumentation method, which may include the application of conversion equations provided by the manufacturer, mathematical models developed in this work, compensation procedures, integrity checks, reading calibration coefficients, or extracting information from communication frames. Finally, the resulting value is corrected using the corresponding linear calibration function before being incorporated into the measurement consolidation process.

Environmental measurements are acquired with georeferencing and time stamping using the information provided by the GNSS receiver. Rather than using a temporal window, the firmware employs a nominal 50 m spatial window to obtain representative measurements for each segment traveled by the Mobile Sensor Node. As shown in Table 2, the longest response time among the sensors that comprise the environmental sensing subsystem is approximately 6 s and corresponds to the particulate matter sensor. Considering an approximate average vehicle speed of 30 km/h under urban conditions, the distance traveled during this interval is approximately 50 m (30 km/h = 8.33 m/s; 8.33 m/s × 6 s ≈ 50 m), which was used as the criterion for establishing the length of the nominal spatial window. This strategy is intended to maintain a constant nominal spatial segmentation, regardless of the time required for the vehicle to traverse each segment or its travel speed. To achieve this, the firmware processes temperature, atmospheric pressure, CO_2_, and solar radiation measurements using a simple moving average. For PM_2.5_, PM_10_, and wind speed, a median filter is applied to remove spikes caused by platform motion or by foreign particles, such as lint, affecting the Honeywell HPMA115S0 sensor (Honeywell International Inc., Charlotte, NC, USA).

If the vehicle increases its speed, not all variables can be updated within the same spatial interval. To ensure that each data frame contains a complete set of environmental variables, whenever a sensor is unable to produce a new measurement within the 50 m segment, the firmware automatically uses the last valid available value under the assumption that, over short intervals, the previously observed environmental conditions have a high probability of remaining unchanged. This strategy preserves the continuity of the records and keeps them structurally verifiable, even when a variable is undersampled.

Once the information has been consolidated, the firmware constructs the data frame and stores it temporarily in local memory. Transmission takes place only when the Mobile Sensor Node establishes communication with a Sink Node. During this process, the node waits for an acknowledgment or rejection message for each transmitted data frame before sending the next one. If the Sink Node is unavailable, the data frames remain stored locally until communication is reestablished.

The graphical user interface remains active throughout system operation, displaying in real time the status of the sensors, the connection to the Sink Node, the start and end coordinates of the measurement interval, the distance traveled, the instantaneous measurements, the calculated average values, and the status of data frame transmission.

Finally, to ensure continuous operation and prevent process blocking, the tasks of sensor data acquisition, processing, storage, communication, and graphical user interface management are executed concurrently using independent threads.

#### 3.2.2. Sink Node Components (Fog Node)

The Sink Node connects the Mobile Sensor Nodes to the Monitoring Center. In addition to acting as a gateway, it is an active system component that ensures system reliability by implementing mechanisms for data integrity validation, duplicate removal, persistent storage, automatic failure recovery, reception acknowledgment, and concurrent processing.

The Sink Node hardware has a structure similar to that of the Mobile Sensor Node. However, because it is designed to operate as a stationary unit without environmental sensors or an autonomous power supply, its hardware architecture is simpler. It consists primarily of a Raspberry Pi 4 Model B, which serves as the node’s central processing unit. This single-board computer incorporates Ethernet and WiFi communication interfaces used to establish the connection with the Monitoring Center through a wired or wireless network infrastructure. Communication with the Mobile Sensor Node is provided by a Digi XBee PRO S3B RF communications module connected to the Raspberry Pi through a USB-to-UART interface. For the local user interface, a 7-inch capacitive touchscreen and a wireless keyboard were incorporated. The node is powered directly from the electrical grid through a DC power adapter. Finally, all components were housed in a plastic enclosure that provides mechanical protection and incorporates an air extractor to promote ventilation and maintain an adequate internal temperature during operation.

The Sink Node firmware integrates configuration, operation, and monitoring functions through its graphical user interface. Initially, it allows configuration of the IP addresses of the Mosquitto MQTT broker and the MongoDB server, as well as the ID, DH, and DL parameters of the Digi XBee PRO S3B communications module to establish communication with the Mobile Sensor Node. During operation, the node receives the data frames transmitted over the wireless link. Each data frame is validated using a checksum before storage, and frames containing errors or corresponding to duplicate records are discarded. Once this verification has been completed, the node sends either an acknowledgment or a rejection message to the Mobile Sensor Node. The validated data frame is then structured as a JSON document for publication to the MQTT broker and subsequently stored permanently in MongoDB through PyMongo, which automatically performs the conversion to BSON format. While these processes are being completed, the measurements remain temporarily stored in the node’s local memory. If any of these services becomes unavailable, the system retains the records in local storage and automatically attempts to restore the connection before resuming transmission.

Throughout operation, the graphical user interface continuously displays the operational status of the node in real time, facilitating configuration, monitoring, and diagnostic tasks. To prevent process blocking, the tasks of data reception, validation, publication, and storage are executed concurrently using independent threads.

#### 3.2.3. Monitoring Center (Cloud Platform)

The Monitoring Center consists of a distributed software infrastructure that enables the reception, publication, storage, retrieval, and visualization of the environmental measurements acquired by the Mobile Sensor Nodes. Table 3 summarizes the main components of this architecture.

The proposed architecture can be easily extended to production environments by incorporating HTTPS to secure REST services and MQTTS to secure MQTT message exchange. Likewise, the Publish/Subscribe communication model improves system scalability by facilitating the integration of new IoT services, applications, or actuators, which can subscribe to MQTT topics associated with specific environmental variables, geographical locations, or both, without requiring modifications to the architecture.

The web application implements the operating modes described in Table 3. During real-time monitoring, users can select the environmental variables of interest and, for each variable, visualize the geographical location where the measurement was acquired, its value, and the corresponding date and time. In addition, the measurements are automatically classified into quality levels (good, acceptable, and critical) according to specific ranges defined for each environmental variable, facilitating their immediate interpretation. In the historical query mode, the stored measurements can be displayed on an interactive map through georeferenced queries, facilitating the analysis of the spatial distribution and temporal evolution of the environmental variables. The application also allows the measurement history of a specific sensor to be exported as a text file (.txt).

## 4. Results

### 4.1. Functional Prototype

The main outcome of this work is the development of the WSN–IoT-Based Mobile Environmental Monitoring System for Smart Cities.

Figure 5 presents the functional prototype developed using the proposed methodology. The system integrates a Mobile Sensor Node (Edge), a Sink Node (Fog), and a Monitoring Center (Cloud), forming a distributed platform for the acquisition, transmission, storage, and visualization of environmental variables.

Figure 6 shows the exterior of the developed Mobile Sensor Node from different perspectives, highlighting the environmental sensors and other components.

Figure 7 shows the interior of the Mobile Sensor Node, highlighting its main components.

Figure 8 summarizes the functional workflow implemented in the prototype, from environmental data acquisition to data publication and storage. The diagram describes the sequence of operations executed by the Mobile Sensor Node, the Sink Node, and the Monitoring Center infrastructure, integrating data acquisition, temporary storage, wireless transmission, data validation, publication to the MQTT broker, and storage in MongoDB. This operational workflow implements a strategy for continuous, reliable, and fault-tolerant mobile environmental monitoring while preserving data availability, integrity, and consistency throughout the acquisition, transmission, and storage processes. To achieve this, it incorporates three complementary mechanisms.

First, spatial segmentation organizes data acquisition according to the distance traveled, generating one representative measurement for each segment of the route, regardless of the vehicle speed.

Second, a persistent intermediate storage mechanism stores the data frames locally in the Mobile Sensor Node whenever Sink Node coverage is temporarily lost, and in the Sink Node whenever communication with the Monitoring Center is interrupted. Once connectivity is restored at each stage of the communication process, the stored data frames are automatically retransmitted.

Finally, an integrity verification and flow control algorithm based on checksum validation, acknowledgment message exchange, and duplicate data frame detection is implemented. This algorithm verifies the integrity of each data frame before storage, requests the retransmission of corrupted packets, and prevents duplicate records caused by retransmissions, lost acknowledgments, coverage handovers between Sink Nodes, or the simultaneous access of multiple Mobile Sensor Nodes to the same Sink Node.

Together, these mechanisms ensure monitoring continuity and data integrity during the movement of the Mobile Sensor Node.

Figure 9 shows the software architecture of the Monitoring Center, organized into three functional layers: presentation, business logic, and data infrastructure. The architecture implements two fully decoupled communication paradigms to optimize system performance: a publish/subscribe scheme for near real-time monitoring and a request/response scheme for historical queries. This separation prevents database queries from affecting the visualization of real-time measurements, thereby reducing the computational load on the backend and improving the system responsiveness. The presentation layer implements two independent operating modes.

The first corresponds to real-time monitoring, in which the web application establishes a WebSocket connection with the MQTT broker to receive the measurements published by the field nodes and automatically update the information displayed to the user through a traffic-light indicator system based on air quality thresholds. By establishing direct communication between the web browser and the MQTT broker, this mechanism eliminates the need for continuous requests to the application server or the database, thereby reducing the computational load on the backend.

The second mode corresponds to historical analysis and uses a REST API developed in Node.js to query the information stored in MongoDB. This mode enables geospatial searches using the Haversine algorithm to retrieve measurements recorded within a 200 m radius of a point selected by the user on the map, facilitating the spatial analysis of the measurements as well as the bulk download of historical records from each sensor for external processing.

Overall, this architecture optimizes system responsiveness while providing efficient access to the stored environmental information.

### 4.2. Performance Evaluation

The functional performance of the developed prototype was evaluated through unit testing of each of its components, followed by an integrated system test. For this purpose, the interfaces developed for each component were used, and the temporary files generated during the data acquisition, transmission, and storage processes were inspected.

In the Mobile Sensor Node, the configuration interface and the operational mode were evaluated. The RF communication configuration interface was validated through its three operating options: Configure, View Configuration, and Preset Configuration. For this purpose, the Digi XBee module was repeatedly disconnected and reconnected to verify correct device detection and the proper configuration of the wireless communication parameters. In all tests, the system responded as expected.

Next, the sensor connection interface was evaluated by disconnecting and reconnecting the devices connected to the USB and I^2^C buses. The results confirmed that the application correctly detects the absence of communication with the sensors and prevents the operational mode from starting whenever any of them fails to respond.

The interface was also evaluated during the operational mode of the Mobile Sensor Node. It correctly displayed the communication status, data frame transmission, temporary wireless coverage loss, the start and end coordinates of each 50 m spatial segment, the distance traveled, the temporary measurements acquired within each 50 m spatial resolution, and the final data frame generated for transmission.

Figure 10 presents some of the interface screens obtained during the configuration and operation tests of the Mobile Sensor Node.

Subsequently, two test runs were conducted on consecutive days under real urban traffic conditions, characterized by variable vehicle speeds and stops inherent to vehicular traffic, to evaluate system performance during mobile monitoring. The first test run was conducted from the University to a residential location, and the second was conducted in the opposite direction on the following day. Each route covered approximately 5.8 km and lasted approximately 25 min, corresponding to an average travel speed of 13.9 km/h. During both test runs, the vehicle speed was intentionally kept below 30 km/h. A total of 229 spatially referenced records were collected during the two urban test runs. For this purpose, the temporary files generated for each measurement and the stored data frames corresponding to each nominal spatial segmentation were inspected to verify that the recorded information exhibited a complete and consistent structure. The results suggest that the effective update interval of the node is approximately between 6 and 15 s. However, this interval cannot be determined with complete certainty because it depends on the position updates provided by the GNSS receiver and on the waiting periods introduced by the firmware to clear the data bus before a new acquisition. In addition, the firmware does not query all sensors at uniform time intervals, and whenever a sensor does not provide a new measurement, it temporarily retains the last recorded value. Analysis of the recorded data showed that PM_2.5_ and PM_10_ measurements are typically repeated at intervals of approximately 6 s, while the remaining variables continue to be updated. This indicates that the update rate of the PM_2.5_ and PM_10_ measurements determines the effective spatial resolution of the system for these variables. Consequently, considering a nominal spatial segmentation of 50 m, vehicle speeds close to 30 km/h may cause some environmental variables not to provide a new measurement within each segment, whereas speeds above this value result in undersampling and, therefore, a reduction in their effective spatial resolution.

In the Sink Node, the configuration interface for entering the IP addresses of the MQTT broker and the database server, the RF communication configuration interface, and the operational mode were evaluated, as shown in Figure 11. To validate the configuration interfaces, both correct parameters and intentionally incorrect values were entered to verify the establishment of the connection with the Monitoring Center and the RF wireless communication with the Mobile Sensor Node. In all cases, the system responded as expected.

During short test runs conducted within the University, the operation of the temporary storage and retransmission mechanism implemented in the Mobile Sensor Node was also verified. To this end, the Mobile Sensor Node repeatedly lost and regained the wireless communication link by intentionally switching the Sink Node off and on during the test runs. It was observed that, once connectivity was restored, the data frames temporarily stored in the Mobile Sensor Node were successfully retransmitted to the Sink Node and removed from its temporary memory. In addition, the received data frames were inspected to verify that the retransmission process did not generate duplicate data frames in the Sink Node.

Subsequently, in a controlled laboratory test, the connection between the Sink Node and the MQTT broker was intentionally interrupted to validate the intermediate storage mechanism. During this period, it was verified that the received data frames continued to be stored locally in the Sink Node. Once the connection with the Monitoring Center was restored, all stored data frames were successfully published to the MQTT broker and permanently stored in the MongoDB database. Finally, the temporary records stored in the Sink Node were compared with the records persisted in the database, confirming the correspondence between both datasets. All tests were successfully completed, confirming the correct operation of the temporary storage, automatic retransmission, and data synchronization mechanisms implemented in the system.

Finally, the web application was evaluated by interacting with its two implemented operating modes: near real-time monitoring through WebSocket (IoT) and historical queries through the geovisor. The data acquired during the two urban test runs described above were subsequently used to verify their retrieval and spatial visualization through the geovisor. In addition, the file download mechanism and the 200 m spatial filter implemented in the geovisor for retrieving measurements recorded in the vicinity of a user-selected location on the map were verified. The tests confirmed the correct visualization of real-time measurements, the updating of information in the geovisor, retrieval of historical records, application of the spatial filter, recovery of nearby measurements, and generation of downloadable files. The correct operation of these functionalities also confirms the proper establishment of communication with the MQTT broker and access to the MongoDB database.

Figure 12 presents the main interfaces of the web application used during the evaluation. All evaluated functionalities responded successfully.

Based on the results of the functional tests presented above, the technology readiness level achieved by the prototype was assessed using the Technology Readiness Level (TRL) criteria established by NASA [56] as a reference framework. The environment used for the tests was representative of the intended operating conditions of the system. The Mobile Sensor Node prototype was installed in a vehicle and evaluated during test runs on urban roads under real traffic conditions, while the Sink Node prototype was installed at a fixed location under conditions representative of a road intersection. Likewise, the applications, application server, and database were deployed and put into operation, enabling the integrated operation of the hardware, software, and communication components that comprise the system. Thus, the complete prototype was functionally evaluated in an environment relevant to its intended application. Table 4 presents the assessment of the prototype against the criteria corresponding to TRL 6.

The assessment of the three subsystems indicates that they are consistent with the criteria established for TRL 6. Therefore, the integrated system as a whole achieves a Technology Readiness Level of TRL 6.

### 4.3. Metrological Performance Evaluation

To evaluate the metrological performance of the developed sensor node, the measurements obtained were compared with those recorded by the reference station during the experimental campaign described in Section 2. Based on this comparison, the calibration functions for each environmental variable were first obtained, and subsequently, the metrological performance metrics were calculated for each environmental variable.

Simple linear regression was used to estimate the calibration function for the temperature, barometric pressure, wind speed, solar radiation, and PM_2.5_ and PM_10_ particulate matter sensors. Table 5 summarizes the errors of the uncalibrated measurements and the parameters of the resulting calibration models (slope and intercept) for each environmental variable.

Figure 13 presents the relationship between the measurements obtained by the node sensors and the values recorded by the reference station, together with the corresponding linear regression models and their coefficients of determination.

The coefficients of determination indicate that some variables, such as wind speed and solar radiation, are well described by the linear model (R^2^ > 0.9). However, for other variables, such as particulate matter and barometric pressure (R^2^ ≈ 0.7), the model exhibits greater uncertainty, suggesting that simple linear regression is not the most appropriate approach.

The Mean Normalized Bias values show that several sensors exhibited systematic deviations from the reference station before the calibration functions were applied. Temperature and particulate matter measurements tended to overestimate the reference values, whereas the global solar irradiance sensor showed a tendency to underestimate them. These differences highlight the need to apply variable-specific calibration adjustments.

In the case of temperature, the observed overestimation may be associated with the additional heating produced by solar radiation incident on the node and by the heat dissipation of the sensor’s internal electronic components. It should also be noted that the MPL115A2 is primarily a barometric pressure sensor that uses temperature to compensate its own pressure measurements and is therefore not optimized as a dedicated ambient temperature sensor. Future prototypes should incorporate a sensor specifically designed for ambient temperature measurements.

For the HPMA115S0, due to the high bias observed in the raw PM_2.5_ and PM_10_ measurements, the possibility of a temporal mismatch between the records from the node and those from the reference station was initially examined. For this purpose, the timestamps and values of the other environmental variables simultaneously acquired by the node and compared with the UNIMAG KOICA station were reviewed. This review did not reveal a shift in the node timestamps, since the other variables maintained temporal correspondence with the records from the reference station. Therefore, no indication was found that the high PM bias was associated with a temporal shift in the records. The overestimation of particulate matter concentrations may be related to factors inherent to the optical measurement principle, including particle accumulation and adhesion within the sensing chamber, as well as the influence of environmental conditions. Regarding sensor fouling, the sensor inlet was inspected and was clean at the beginning of the calibration, whereas dust residues were observed at the end of the measurements. Although this observation indicates that particle accumulation occurred during the experimental period, it does not constitute sufficient evidence to attribute the high observed bias to this factor. In coastal environments such as Santa Marta, high relative humidity may promote the hygroscopic growth of suspended particles, altering their size and optical properties and increasing the amount of scattered light detected by the sensor [28]. However, detailed information on the measurement principle and detection element, as well as on the conditioning, compensation, or processing procedures used by the UNIMAG KOICA station for PM_2.5_ and PM_10_ measurements, is not available. Consequently, it is not possible to determine whether these conditions affect both systems equivalently, which should be considered when interpreting the observed bias. Because relative humidity was not included in the regression model, this potential influence represents a limitation of the calibration performed. Future prototypes should incorporate periodic sensor inspection and cleaning protocols, as well as multivariable calibration models that include meteorological variables, particularly relative humidity and wind speed.

In the case of solar radiation, the RK200-04 is not a high-precision thermopile pyranometer (Class A/B according to ISO 9060 [57]), but rather a silicon-based sensor with a limited spectral response (300–1100 nm). As a result, it cannot capture the full radiant power of the incident solar energy because part of the solar spectrum lies outside its spectral response range, thereby affecting the measurement.

For the CO_2_ sensor and the developed electronic rain gauge, calibration by linear regression against the reference station was not possible because of the characteristics of the measured variables and the lack of directly comparable reference data.

The Atlas Scientific EZO-CO_2_ sensor, which is based on non-dispersive infrared (NDIR) technology and is classified by the manufacturer as a research-grade sensor, was not experimentally calibrated against a reference but was instead used with its factory calibration. This decision was made because no reference CO_2_ data were available: the UNIMAG KOICA station does not measure this variable, and a certified calibration gas cylinder was not available. Nevertheless, the sensor performance was evaluated through a statistical analysis of its temporal stability and repeatability using the hourly sensor readings. For each measurement interval, the mean (664.532 ppm), standard deviation (<9 ppm), and coefficient of variation (<1.3%) were calculated, yielding satisfactory results in terms of sensor consistency, as shown in Figure 14.

For the self-developed electronic rain gauge, experimental calibration against an external reference was also not performed. This was because the device output classifies precipitation into three levels based on the flow rate through a small outlet orifice, whereas the UNIMAG KOICA station reports accumulated precipitation (mm). These differences in the physical nature of the measurements prevent a direct comparison between the two systems. However, the subsystem was supported by a previous hydrodynamic flow calibration analysis that reported a relative error of 8.5% and, based on the measurements reported in that study, an RMSE of 5.16 mm [46]. Because these results were obtained under different experimental conditions and no directly comparable reference measurements were available during the present field campaign, the NRMSE was not estimated for the rainfall subsystem.

After applying the calibration functions, the RMSE, MNB, and NRMSE were recalculated for each environmental variable. Table 6 presents the resulting values and enables a comparison of the metrological performance of the different sensors after calibration. Overall, a reduction in error was observed for most of the evaluated variables.

After applying the calibration functions, the RMSE, MNB, and NRMSE were recalculated for each environmental variable using the calibration dataset. Subsequently, the same calibration functions were applied, without refitting, to the independent validation dataset, and the RMSE, MNB, and NRMSE were calculated for these data. Table 6 presents the resulting values and enables a comparison of the metrological performance of the different sensors after calibration for both the calibration dataset and the independent validation dataset.

After applying the calibration functions, the RMSE, MNB, and NRMSE were recalculated for each environmental variable using the calibration dataset. Table 6 presents the resulting values and enables an evaluation of the performance of the calibration process. Subsequently, the same calibration functions were applied, without refitting, to the independent validation dataset. The RMSE, MNB, and NRMSE were calculated before and after calibration using the validation dataset, and the resulting values are presented in Table 7.

The independent validation results show that the calibration functions maintained favorable performance for four of the six variables evaluated. The greatest improvement was observed for PM_2.5_, for which the RMSE decreased from 54.367 to 8.284 µg/m^3^, followed by global irradiance, with a reduction from 75.466 to 61.082 W/m^2^. Reductions in RMSE were also observed for PM_10_, from 12.561 to 11.416 µg/m^3^, and barometric pressure, from 1.575 to 1.481 hPa. In contrast, temperature and wind speed showed increases in RMSE from 0.400 to 1.667 °C and from 1.038 to 1.250 m/s, respectively. These results indicate that, although the calibration functions improved performance for most variables in the independent validation dataset, their effect was not uniform across the sensors, indicating lower generalization capability for temperature and wind speed under the conditions of the second measurement campaign.

Overall, the results show that the metrological performance varies among the evaluated environmental variables. Therefore, the performance of the sensor node is assessed individually for each variable using RMSE, MNB, and NRMSE, rather than through a single aggregated performance index.

## 5. Discussion

The results obtained during the evaluation of the proposed system both in the laboratory and during urban routes demonstrate that the prototype meets the requirements corresponding to Technology Readiness Level 6 (TRL 6). According to the definition of the Technology Readiness Levels, TRL 6 corresponds to the demonstration of a fully integrated prototype operating under representative conditions of the intended application environment, whereas TRL 5 is mainly limited to the validation of integrated components or subsystems in a relevant environment. In this work, the hardware, firmware, wireless communications, IoT infrastructure, and distributed services operated jointly while the system was subjected to mechanical vibrations, variable vehicle speeds, temporary interruptions in wireless connectivity, and environmental conditions typical of the urban environment. These experimental findings support that the prototype satisfies the criteria established for TRL 6. Nevertheless, the system has not yet reached TRL 7 because it has not been validated through prolonged deployment within a fleet of public transportation vehicles, nor has it been subjected to continuous operation over extended periods of time.

Although the system satisfactorily fulfilled the functional requirements defined for this stage of the project, the experimental tests also identified opportunities for improvement aimed at future commercial implementation or large-scale deployment.

During the development of the prototype, several design decisions prioritized ease of construction, debugging, and integration, consistent with an iterative and incremental development process, rather than the robustness required for an industrial product. In particular, the touchscreen installed on the sensor node enclosure significantly facilitated firmware configuration, functional validation, and adjustment activities during development. However, this solution is not suitable for permanent operation on public transportation vehicles because of its vulnerability to vandalism, solar radiation, humidity, and mechanical deterioration. In future versions of the system, this interface could be replaced by a wireless application. Likewise, the charge controller could be relocated inside the enclosure. These modifications would allow the sensor node to remain completely sealed, thereby protecting the electrical connections and electronic components against rain, humidity, dust, and corrosion, while increasing the system reliability during prolonged operation. Additionally, in future versions, it would be advisable to incorporate a wind direction sensor and a compass. The information provided by these sensors could be used to improve the measurement processing and compensation algorithms during vehicle movement. The monitoring capability could also be expanded by incorporating sensors for other atmospheric pollutants of urban interest, such as CO, NO_2_, and O_3_. This redesign corresponds to an industrial evolution of the hardware and does not alter the proposed functional architecture.

The development and testing of the prototype also made it possible to identify practical aspects associated with the use of low-cost sensors that should be considered in subsequent iterations of the system. One of the main limitations is related to their physical construction, since some of these sensors have connectors, enclosures, and levels of environmental protection that are less robust than those used in reference-grade, scientific-grade, or industrial-grade instruments. This aspect is particularly relevant for permanent installation on a mobile platform, where mechanical stress and vibrations may compromise hardware integrity. Likewise, the integration of some sensors required additional hardware and firmware processes due to the characteristics of their communication interfaces and data output formats. Additionally, the measurement principle and the sensing and transduction elements may differ from those used in reference-grade, scientific-grade, or industrial-grade instruments, which may influence characteristics such as spectral response, dynamic range, and operating conditions.

Despite these limitations, the use of low-cost sensors proved suitable for the iterative development of the prototype, as it made it possible to implement, progressively expand, and functionally test the system with a lower investment compared with proprietary environmental data acquisition systems or the use of reference-grade instruments. Rapid prototyping also makes it possible to evaluate sensor performance within the system and, based on the results obtained, determine whether they should be retained or identify the improvements required in subsequent iterations. This flexibility is particularly important when multiple environmental variables need to be monitored, as it allows new sensors to be progressively incorporated according to the application requirements, without relying exclusively on the instrumentation and interface options available in a proprietary data acquisition system.

In contrast to the limitations identified in the hardware, the software architecture was designed to facilitate extensibility and provide a basis for scalability. The separation between the near real-time information flow through MQTT over WebSockets and the historical queries implemented through REST services connected to MongoDB made it possible to completely decouple data acquisition from the storage and visualization processes, avoiding computational bottlenecks during the experimental tests. This organization also facilitates the future incorporation of new sensor nodes, client applications, and IoT services without modifying the overall system architecture. However, the experimental tests did not include load testing or the simultaneous operation of multiple sensor nodes; therefore, system scalability was not experimentally validated and should be considered a potential architectural capability that must be evaluated in future deployments.

Regarding the scope of the experimental evaluation, the prototype was assessed through controlled functional tests and tests under real conditions, each aimed at verifying different aspects of system operation. The urban test runs were used to verify the integrated operation of the system during mobile data acquisition and subsequent retrieval and visualization through the geoviewer. The short test runs conducted within the University enabled verification of temporary storage and data frame retransmission under controlled interruptions of the wireless link between the Mobile Sensor Node and the Sink Node. Likewise, controlled interruptions of the communication between the Sink Node and the MQTT broker enabled verification of intermediate storage and subsequent data synchronization with the database. These tests were primarily designed as functional verifications rather than as a specific characterization of communication performance. Therefore, metrics such as per-frame end-to-end latency, number of retransmissions, packet loss rate, and precise link recovery time were not included within the scope of the evaluation performed. Obtaining these metrics would require incorporating additional logging mechanisms into the node firmware or external monitoring tools, which represents a relevant aspect for a more comprehensive quantitative evaluation in future deployments.

The current implementation of the system presents security limitations that must be addressed before operational deployment. In particular, the prototype does not implement comprehensive mechanisms for device and user authentication, access control, or secure device-to-broker communication. These requirements should be addressed by incorporating MQTTS for communication with the MQTT broker and HTTPS for REST services, together with appropriate authentication and authorization mechanisms.

An additional limitation is related to the validation of measurements during vehicle movement. The calibration functions were obtained with the Mobile Sensor Node positioned stationary next to the UNIMAG KOICA fixed reference station, which made it possible to temporally unify the measurement intervals and compare the variables recorded by both systems. However, the metrological performance during movement was not experimentally evaluated, because the reference station provides records with a temporal resolution of one hour, while the Mobile Sensor Node generates measurements according to the spatial segmentation of the route. Even when performing routes in the vicinity of the reference station, the difference in the temporal resolution of both systems makes it difficult to establish a direct correspondence between their measurements. This limitation is particularly relevant for variables with high temporal variability, such as wind speed and solar radiation, which may present significant changes over short intervals. For PM_2.5_ and PM_10_, in addition to temporal variability, spatial variability must be considered, since during movement the Mobile Sensor Node may record local conditions different from those observed by the fixed reference station. In this case, a comparison with fixed stations would require considering appropriate temporal and spatial aggregation windows to obtain more representative values, although this would reduce the spatial resolution provided by mobile monitoring. On the other hand, during movement, wind speed measurement is affected by vehicle speed and airflow direction, although compensation was implemented using the speed obtained through GNSS. A more appropriate validation under mobility conditions would require two equivalent and previously calibrated sensor nodes, one operating in a stationary position and the other installed on the vehicle, with simultaneous acquisition of measurements. This procedure would make it possible to experimentally evaluate the effect of mobility on the different environmental variables and, particularly for wind speed, the effectiveness of the implemented compensation.

The results obtained during the metrological evaluation reveal opportunities to incorporate Artificial Intelligence (AI) techniques into future versions of the system. These research directions arise directly from the limitations observed during the experimental evaluation and not solely from the recent advances in these technologies. First, the incorporation of machine learning models would replace the simple linear regression used during calibration with multivariable models capable of simultaneously considering other meteorological variables that particularly affect the behavior of low-cost sensors. This would reduce the observed errors, especially in the estimation of PM_2.5_ and PM_10_ under coastal environments such as Santa Marta. Second, the results obtained during mobile monitoring showed that the nominal 50 m spatial segmentation may result in undersampling of some variables as vehicle speed increases, due to the different sensor update times. For a subsequent development iteration, increasing the nominal spatial window to 100 m could be evaluated, which would allow higher vehicle speeds to be considered in relation to the observed update times. As a further evolution, the incorporation of Artificial Intelligence techniques would make it possible to replace a fixed spatial segmentation with adaptive models capable of automatically determining the most appropriate spatial resolution according to vehicle speed, sensor response times, and the environmental variability of the surrounding conditions. In this way, the spatial resolution would no longer be a fixed parameter but would become a dynamic variable dependent on the actual operating conditions. Additionally, specific algorithms could be incorporated for each variable instead of the current methods based on the mean and the median to consolidate the measurements within each spatial segment.

Finally, the geo-viewer could evolve from the current scheme based on spatial filters toward intelligent geospatial reconstruction models. In this context, deep learning techniques or advanced spatial interpolation models would facilitate the analysis of the measurements obtained along multiple routes to construct spatial gradients of the monitored environmental variables, generating complete city maps that support urban planning, environmental management, and decision-making within the framework of smart cities.

## 6. Conclusions

The implementation of a mobile environmental monitoring system, composed of low-cost sensors and integrated through an Edge–Fog–Cloud architecture interoperable with IoT services, demonstrated the feasibility of extending the spatial coverage of urban environmental monitoring in smart cities while requiring less technological infrastructure than traditional schemes based exclusively on fixed monitoring stations, providing georeferenced environmental measurements with a nominal spatial segmentation of 50 m. The designed and implemented methodology proved suitable for the progressive development and integration of complex hardware and software subsystems, facilitating the functional validation of the complete system. The Edge–Fog–Cloud functional architecture, based on WSN and IoT, decoupled the functions of data acquisition, storage, retrieval, and visualization, providing extensibility and a basis for potential scalability by facilitating the incorporation of additional sensor nodes, client applications, and IoT services without modifying its functional structure. The functional evaluation confirmed that the prototype achieved Technology Readiness Level (TRL) 6. The metrological evaluation using independent validation data showed that the calibration functions reduced the RMSE for four of the six variables evaluated against the reference station, with the greatest improvement observed for PM_2.5_. However, the increased RMSE for temperature and wind speed showed that calibration performance varies among sensors and should be evaluated individually for each environmental variable. The evaluations conducted also identified the main lines of action for continuing the development of the prototype toward higher Technology Readiness Levels. These include the quantitative evaluation of the system with a larger number of sensor nodes and in larger-scale deployments, as well as improving the calibration of variables that showed lower generalization capability. In this regard, the metrological results guide future work toward the incorporation of Artificial Intelligence techniques for processing and calibrating the raw measurements acquired by the sensors. In addition, adaptive spatial processing and geospatial reconstruction using Artificial Intelligence constitute future development lines for strengthening the capabilities of the mobile environmental monitoring system.

## Figures and Tables

**Figure 1:**
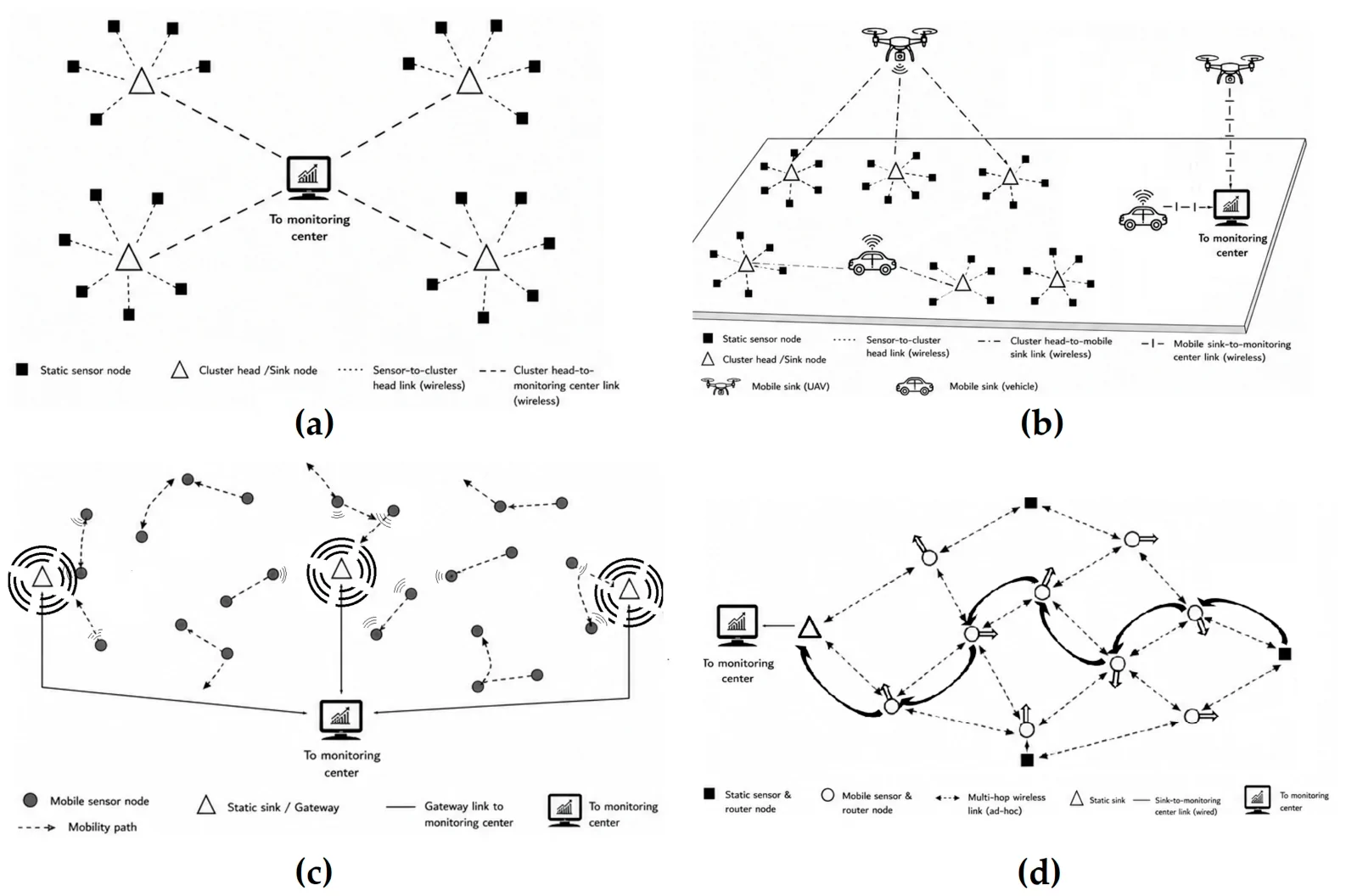
Topologies of Wireless Sensor Networks (WSNs): (**a**) Static Hierarchical WSN; (**b**) WSN with a Mobile Sink; (**c**) WSN with Mobile Sensor Nodes and Static Sinks; (**d**) WSN as a Mobile Ad Hoc Network.

**Figure 2:**
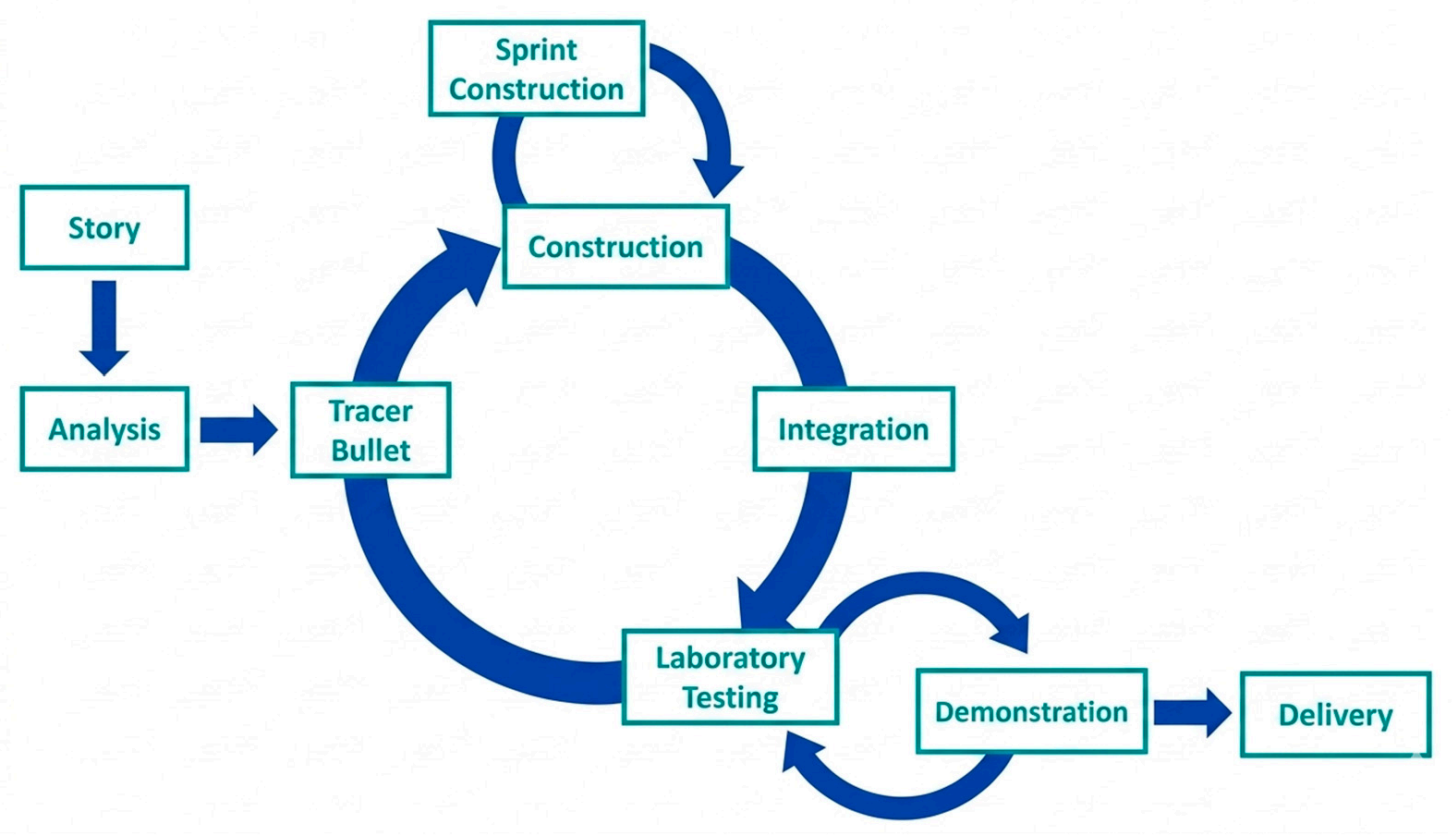
Proposed iterative and incremental development methodology.

**Figure 3:**
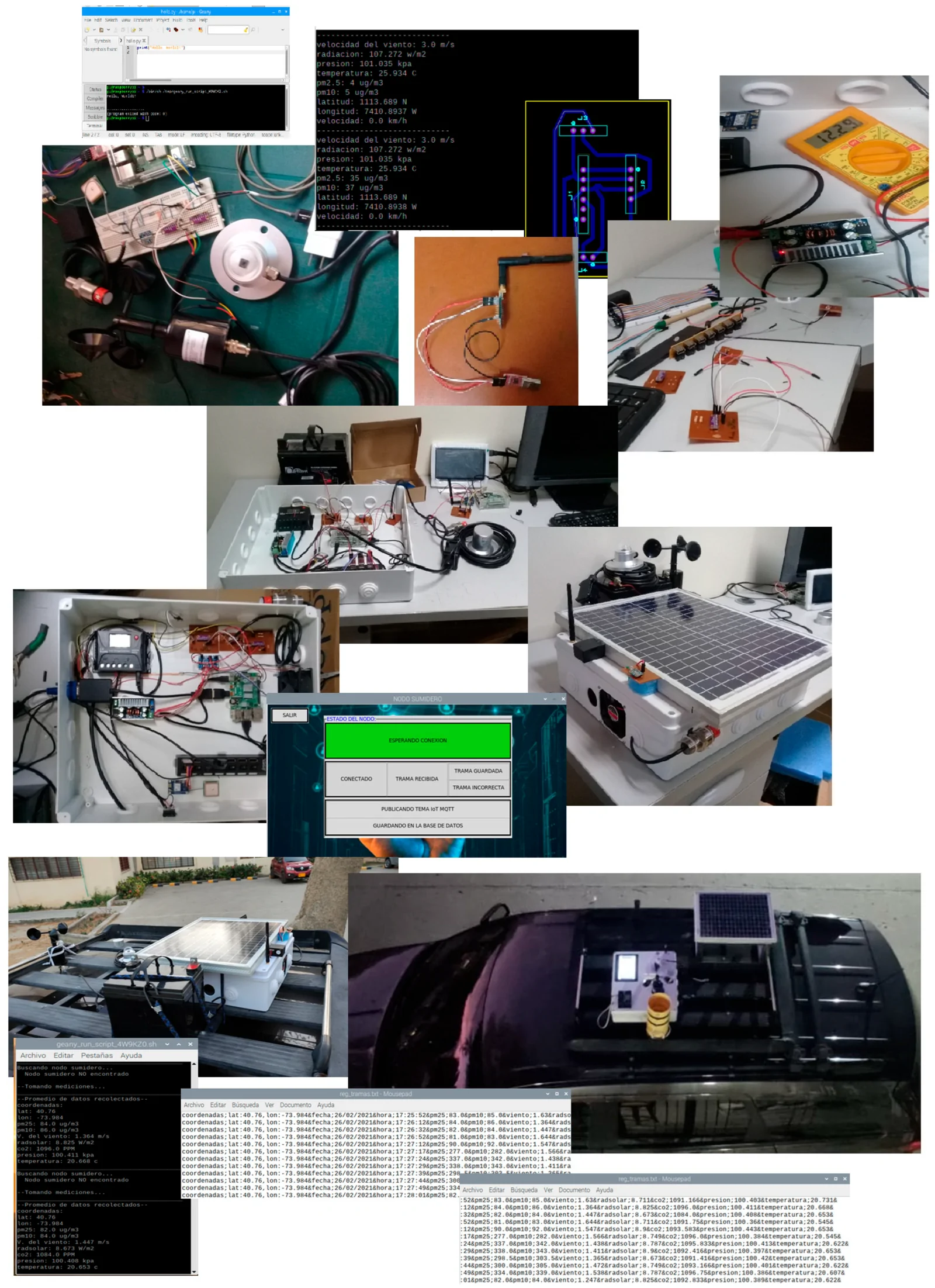
Incremental development of the Mobile Sensor Node.

**Figure 4:**
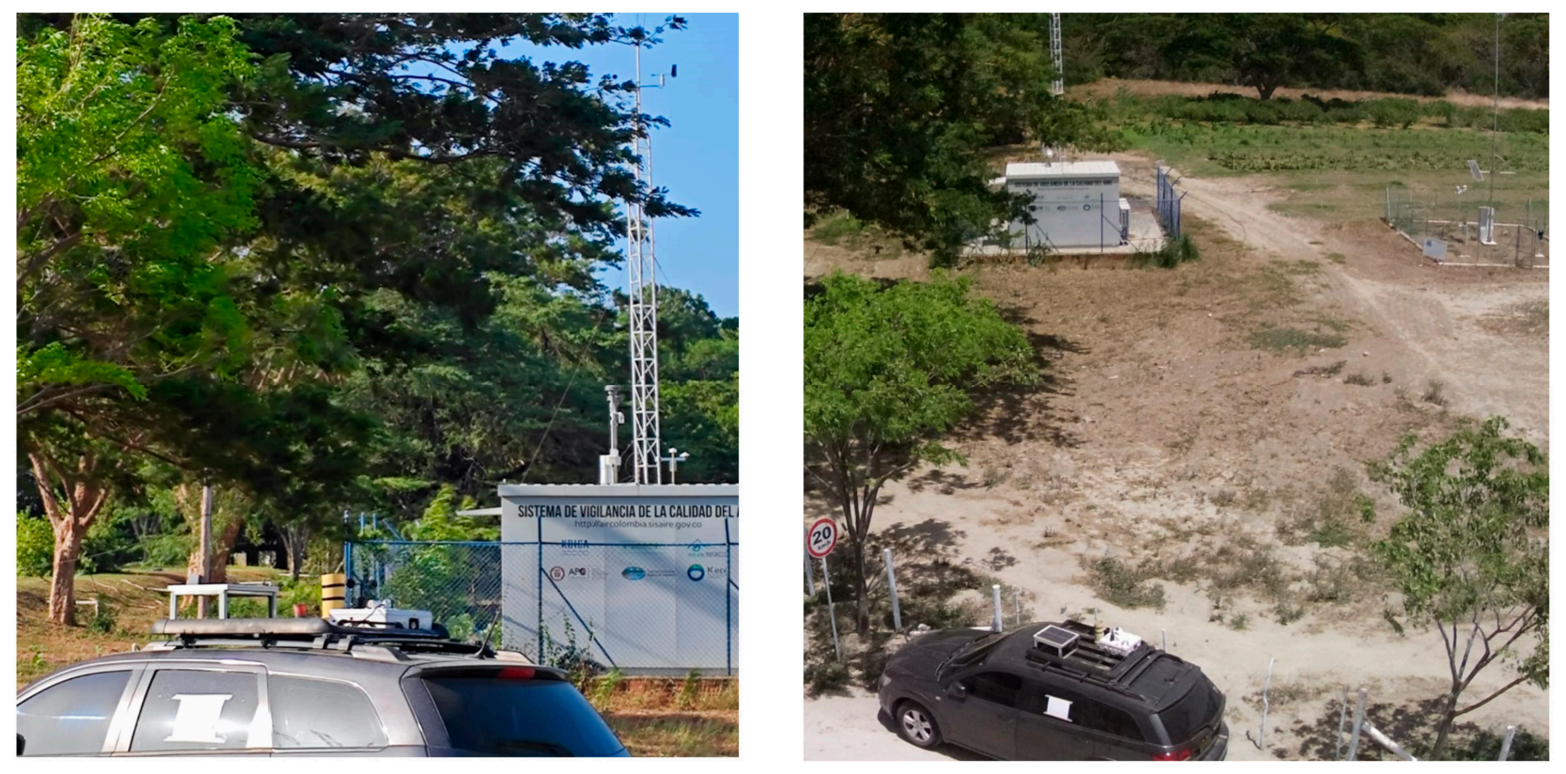
Experimental setup for co-location calibration.

**Figure 5:**
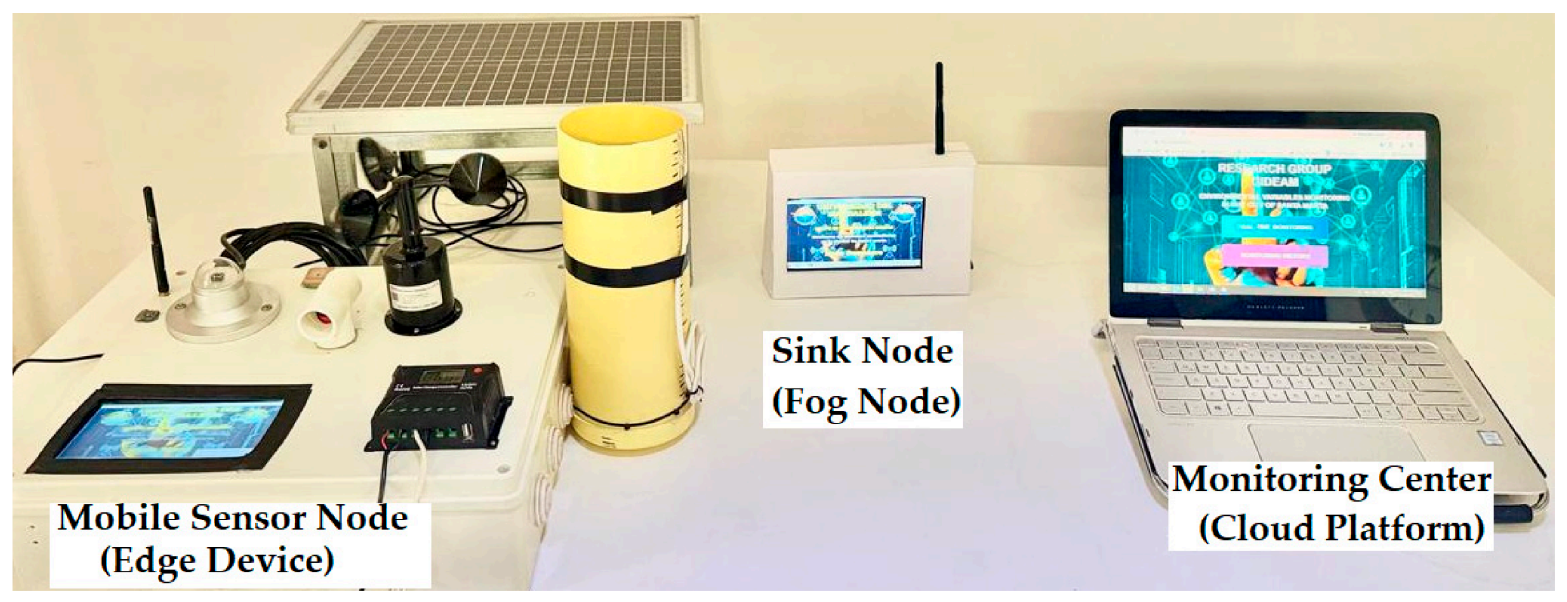
WSN–IoT-Based Mobile Environmental Monitoring System.

**Figure 6:**
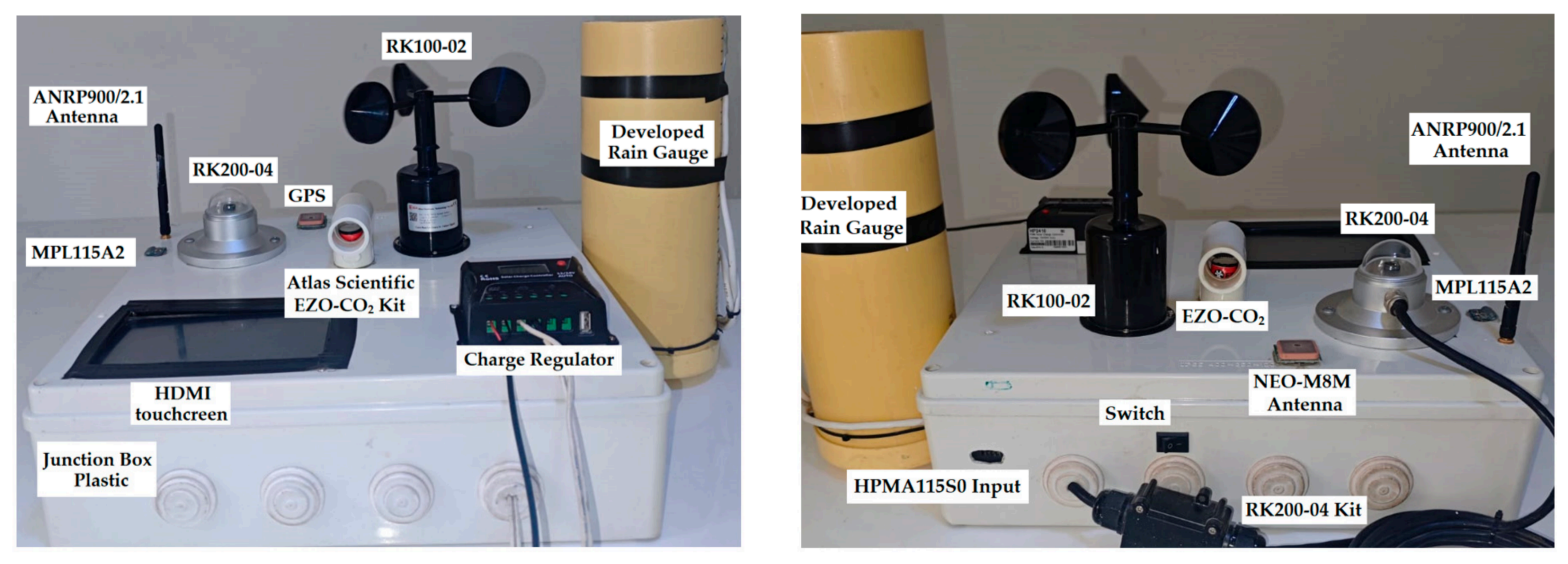
Photographs of the exterior of the Mobile Sensor Node.

**Figure 7:**
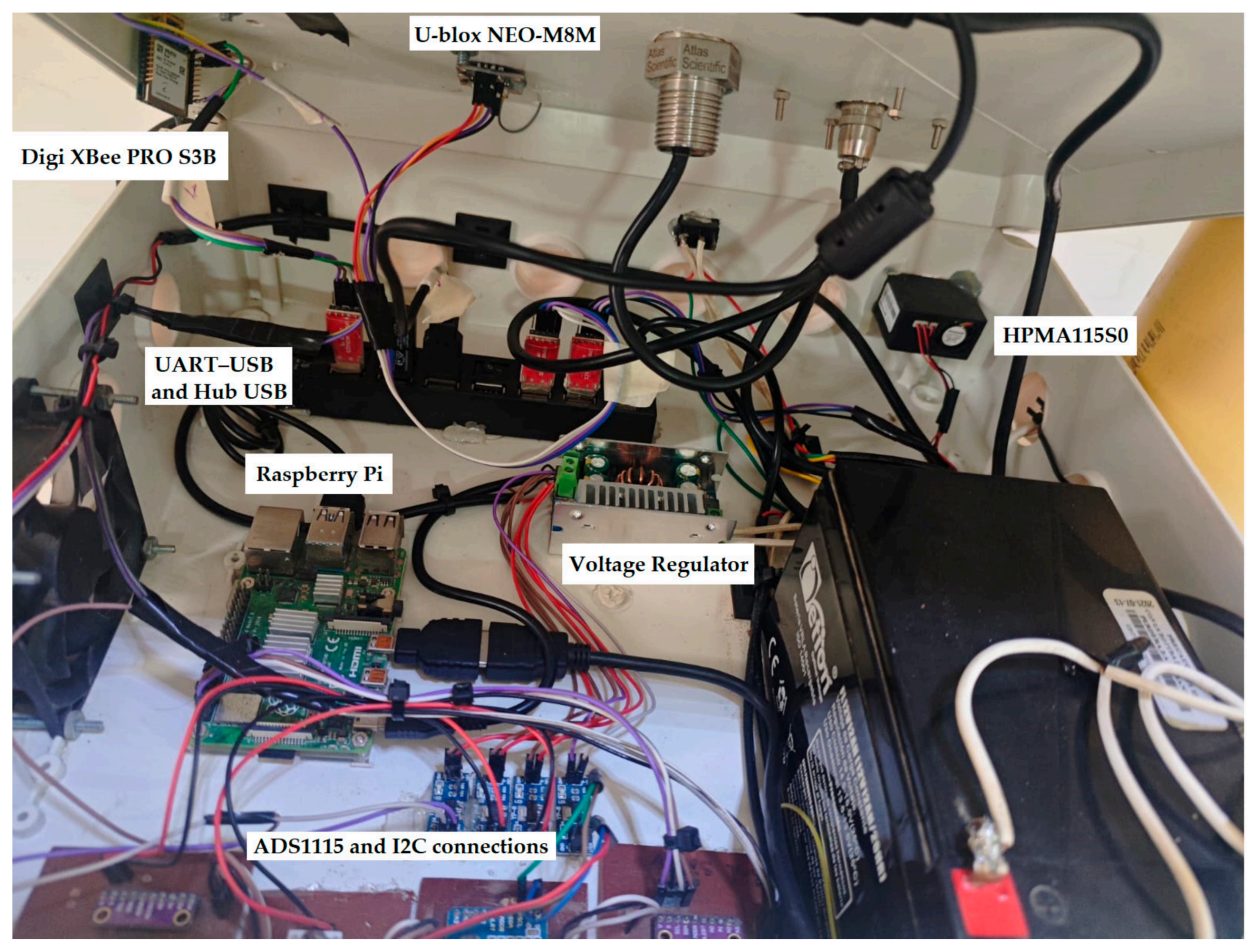
Photographs of the interior of the Mobile Sensor Node.

**Figure 8:**
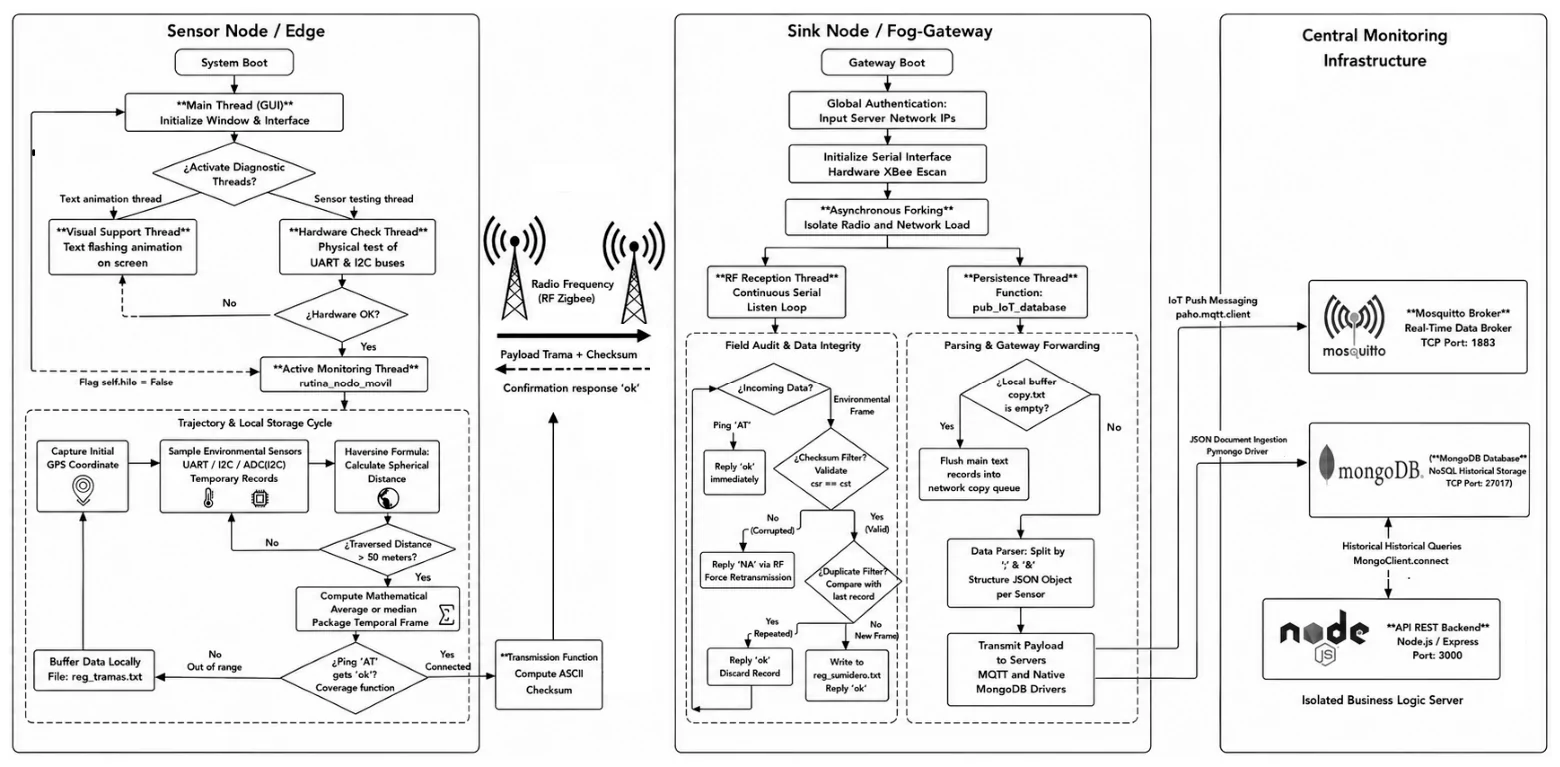
Functional workflow of the Sensor Node and Sink Node. ** indicates main threads, functions, or external system components.

**Figure 9:**
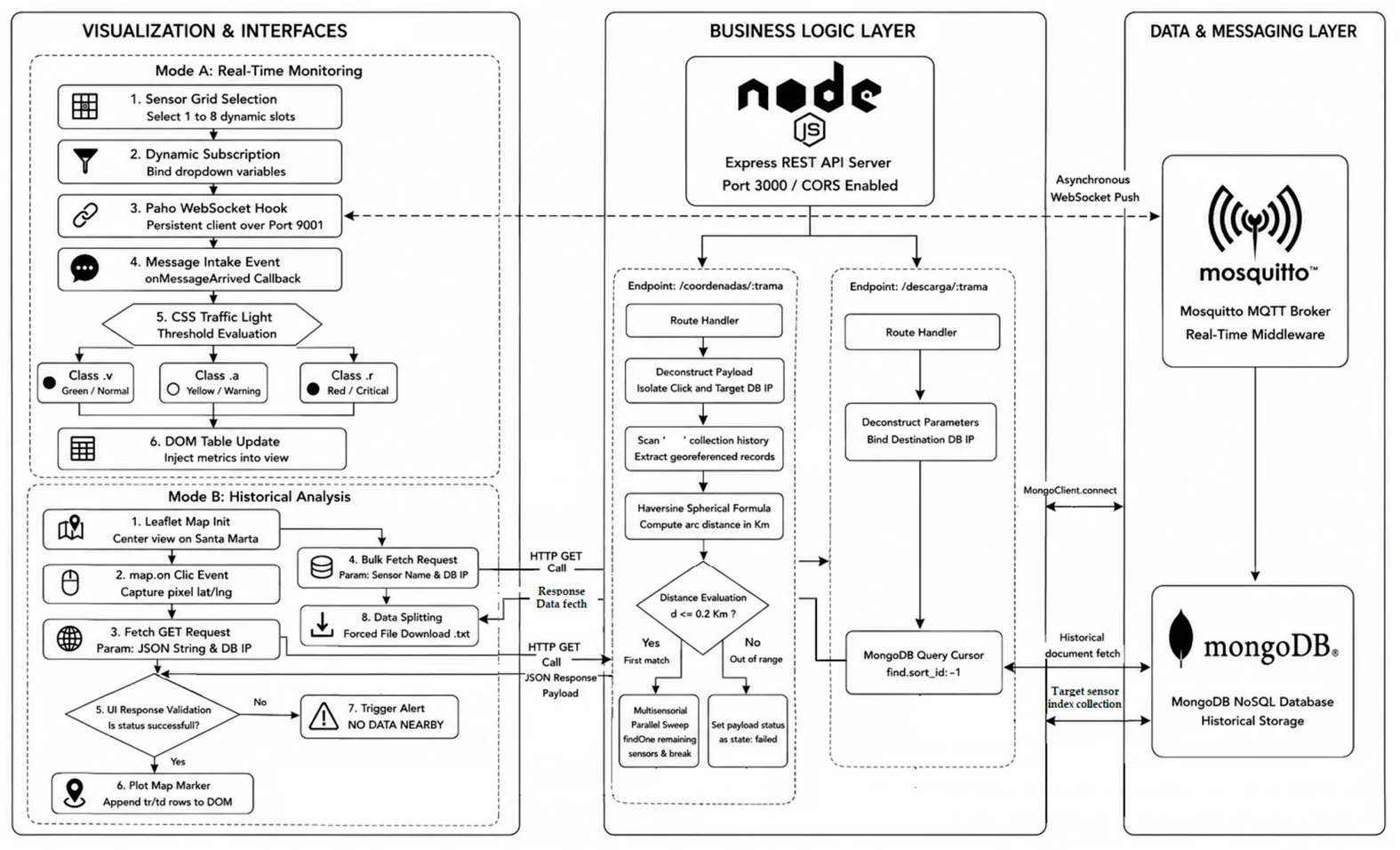
Software architecture of the Monitoring Center.

**Figure 10:**
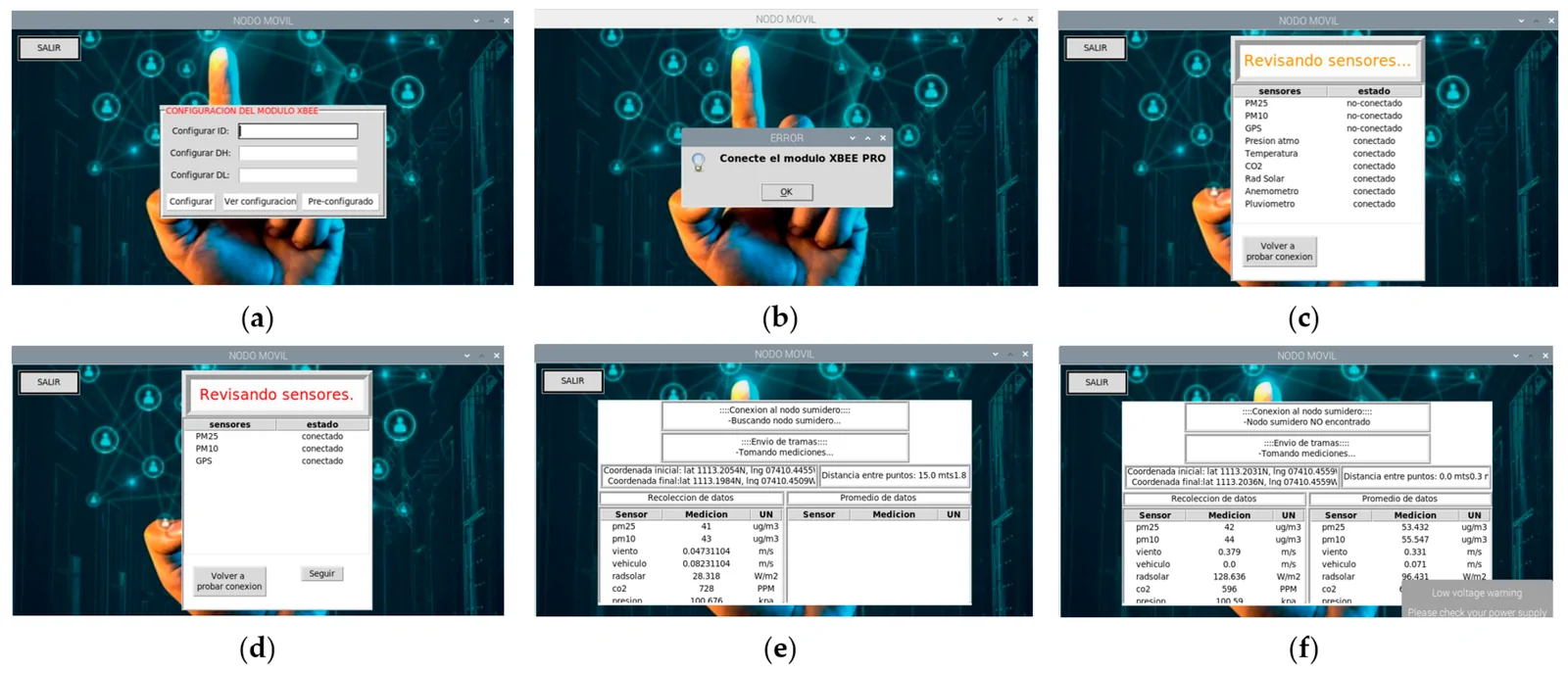
Screenshot Mobile Sensor Node: (**a**,**b**) RF communication configuration tests; (**c**,**d**) sensor connection tests; (**e**,**f**) node operation test.

**Figure 11:**
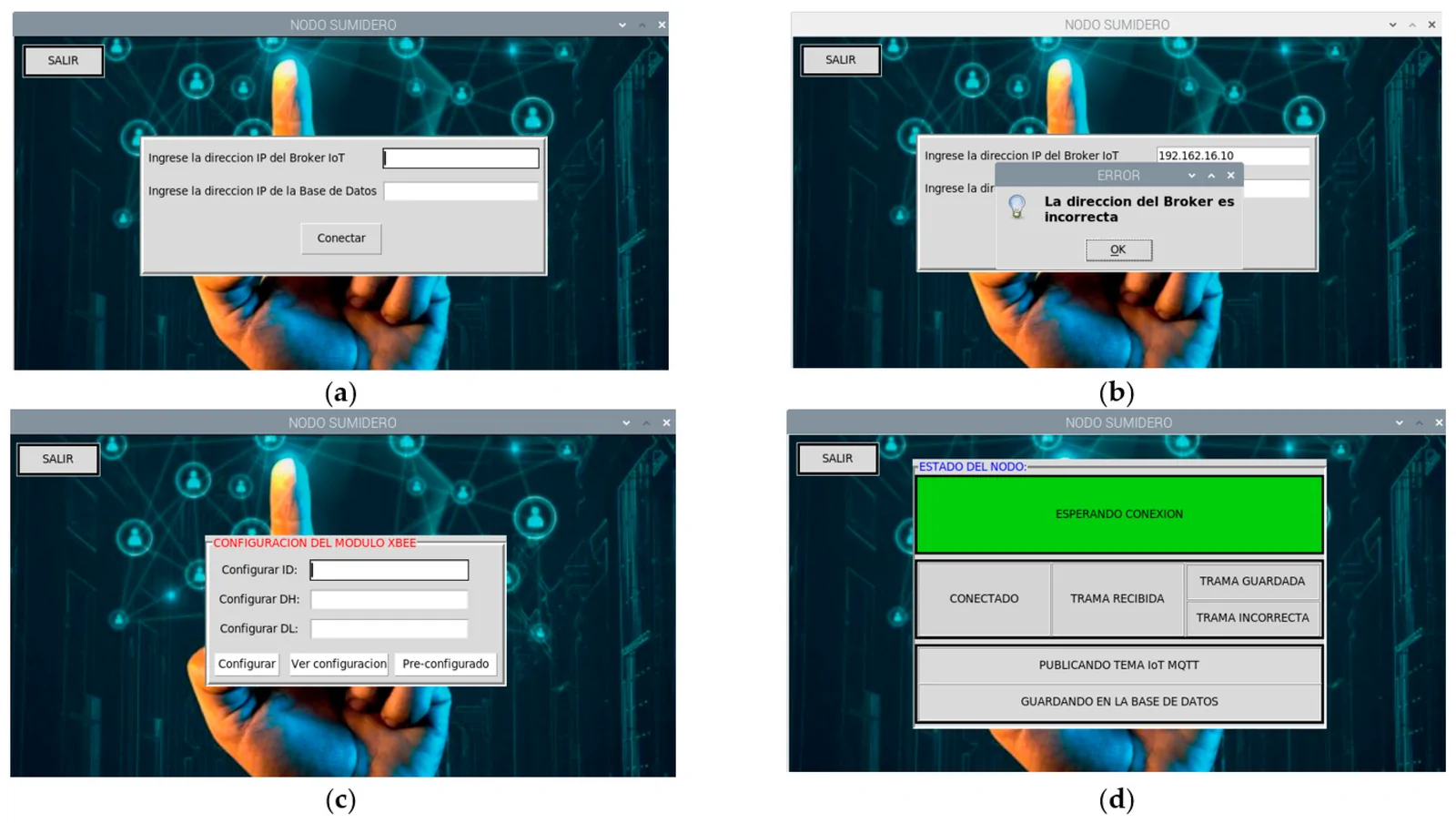
Screenshot Sink Node: (**a**,**b**) IP configuration and monitoring center connection tests; (**c**) RF communication configuration tests; (**d**) node operation test.

**Figure 12:**
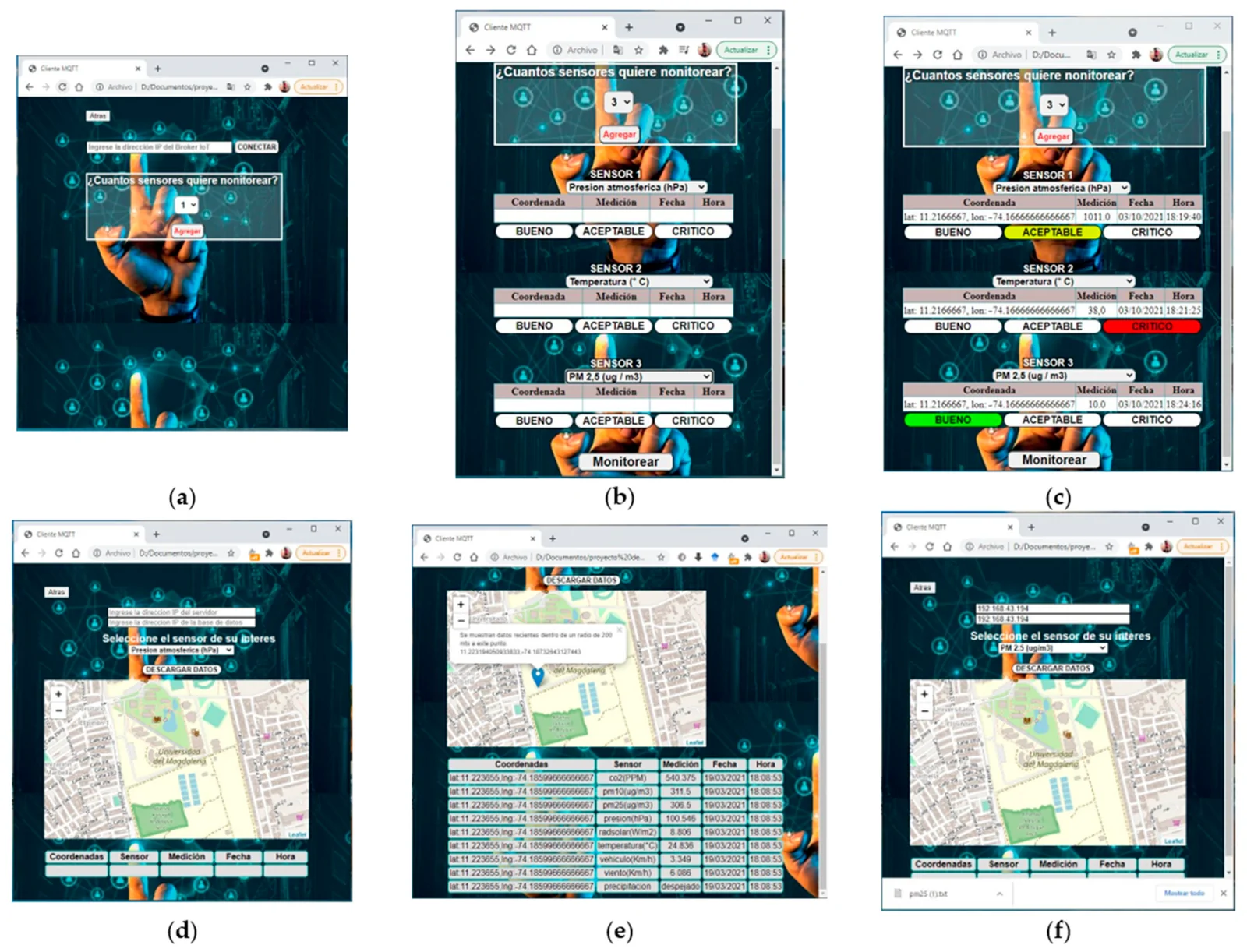
Screenshot of the web application: (**a**–**c**) near real-time monitoring tests using WebSocket (IoT); (**d**–**f**) geoviewer tests for file download and the 200 m spatial filter.

**Figure 13:**
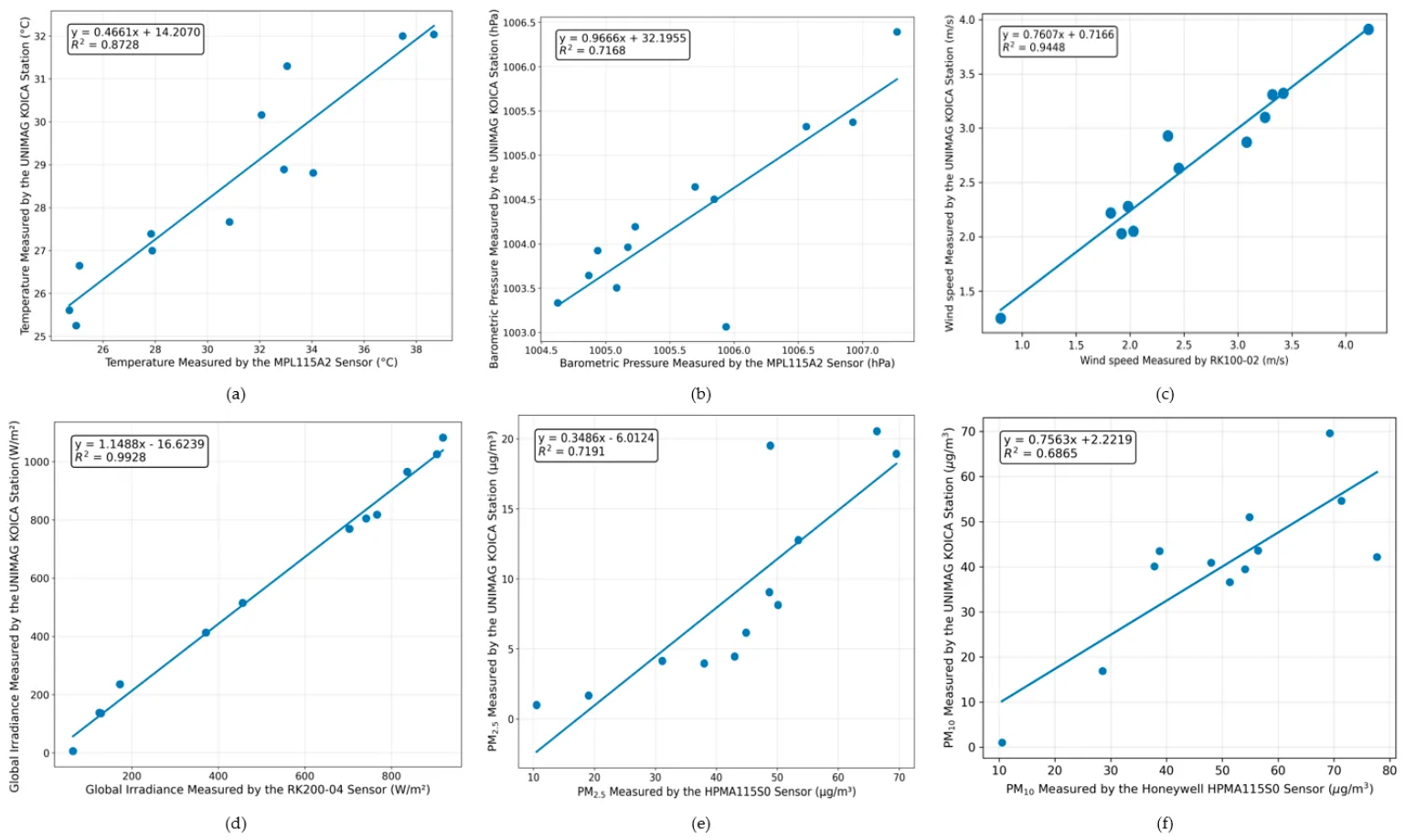
Linear regression: (**a**) Temperature; (**b**) Barometric Pressure; (**c**) Wind Speed; (**d**) Global Irradiance; (**e**) PM_2.5_; (**f**) PM_10_.

**Figure 14:**
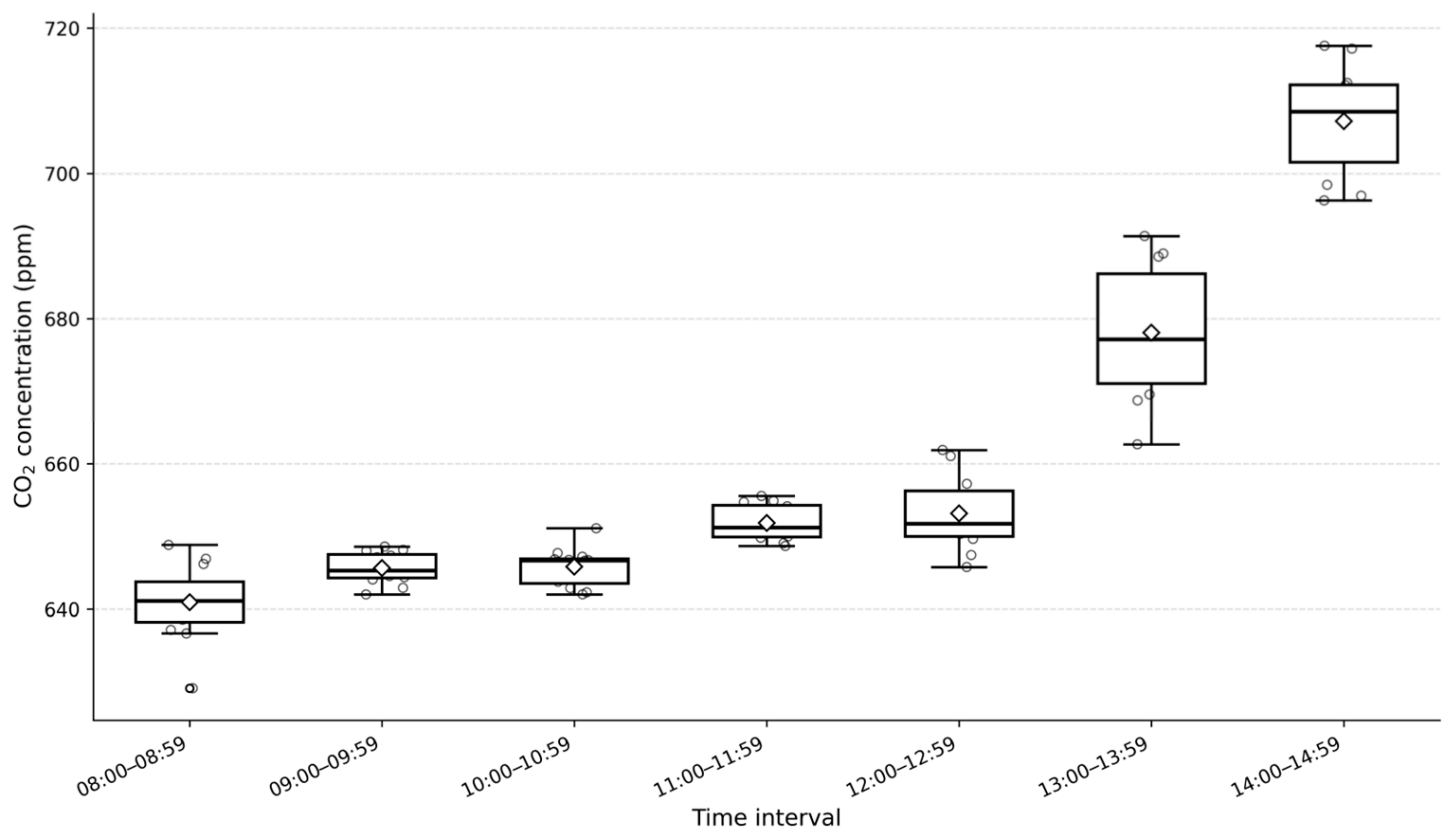
Boxplot for CO_2_ measurements taken for each hourly interval.

**Table 1:** Comparison of mobile environmental monitoring systems.

Ref.	Objective/Sensed Variables	Architecture and Communication Technologies	Data Correction and Synchronization	Hardware/Software Prototype	Experimental Validation
[24]	Perform vehicular microclimate monitoring using a smaller number of sensing devices/Variable: CO_2_.	Vehicular sensor with direct communication to the monitoring server (without a sink node or Fog). ZigBee for intra-vehicle communication between the CO_2_ sensor, located outside the vehicle, and the processing unit inside; GSM/SMS for direct communication with the server through the cellular network.	The measurements are averaged, associated with the vehicle location, and transmitted to the server. No calibration or data transport reliability mechanisms are described.	A prototype is implemented in 16 vehicles, but no development methodology for its hardware and software is described.	A deployment is carried out in Hsin-Chu, Taiwan, demonstrating CO_2_ monitoring over an area of approximately 80 hectares. No validation against reference instruments is reported.
[28]	Identify portable sensor systems capable of reliably quantifying dynamic exposure gradients in urban environments/Variables: PM_2.5_, PM_10_, NO_2_, and BC.	No unified communication architecture is proposed; each portable system uses its own storage and/or transmission mechanisms. For example, SODAQ transmits data to the Cloud via GPRS/4G.	Calibration and evaluation through laboratory tests, co-location with reference instruments, and field tests. The influence of different operating conditions on sensor performance is analyzed. No reliability mechanisms to prevent packet loss during data transmission are described.	No prototypes are developed and no development methodology is presented; commercial sensor systems are evaluated. For the BCmeter, the need for hardware/software developments to convert it into a portable system is identified.	Laboratory tests, a co-location campaign with a regulatory reference station, and a mobile measurement test using a cargo bicycle along an urban route are conducted.
[47]	Evaluate the performance of air quality measurement instruments installed on mobile platforms using fixed regulatory stations/Variables: O_3_, NO_2_, and NO. Derived variable: O_x_ = (O_3_ + NO_2_).	Mobile nodes based on the Aclima measurement and acquisition platform, installed on vehicles, are used. The article does not describe the architecture or communication technologies used for data transmission; however, Aclima reports support for communication through cellular networks and Cloud software for data processing.	Stationary and in-motion comparison with fixed reference stations; temporal averaging and statistical evaluation of measurement bias and performance. For measurements acquired in motion, the distance to the station and road type are considered. No calibration method based on these comparisons is proposed, nor are mechanisms described to prevent data loss associated with measurements acquired in motion or communication.	No hardware/software prototypes are developed and no methodology for their development is presented; mobile measurement platforms previously developed by Aclima are used.	Mobile measurement campaigns are conducted in Denver and the San Francisco Bay Area, comparing the measurements obtained with those from fixed regulatory stations.
[48]	Analyze and optimize the spatio-temporal observability of a mobile urban sensing system/Variables: temperature, relative humidity, and CO_2_.	Edge, Fog, Cloud architecture. Sensor nodes installed on public service vehicles with low-cost sensors, GPS, Raspberry Pi, and LoRaWAN communication toward the Fog layer; the technology and communication protocol from Fog to Cloud are not specified.	Preprocessing at the Edge by smoothing the measurements using a time-sensitive exponentially weighted moving average (EWMA); temporary storage to mitigate losses due to LoRaWAN duty-cycle limitations and areas without coverage. At the Fog, spatio-temporal discretization into cells of approximately 100 × 100 m, aggregation, and local interpolation are performed. In the Cloud, the results from the different Fog domains and the analyses are integrated (Spatio-temporal interpolation). No calibration of the sensors against reference instruments is reported.	Mobile sensor node prototypes are implemented, and software is developed for data acquisition, preprocessing, storage, and distributed processing. No specific hardware/software development methodology is described. The hardware/software implementation of the Fog and Cloud layers is not detailed.	An experimental deployment is carried out in Benevento, Italy, using three vehicles equipped with mobile sensor nodes to validate the spatio-temporal observability and interpolation framework. No validation of the measurements against reference instruments is reported.
Proposal	Develop a multivariable mobile system for urban environmental monitoring with spatial resolution/Variables: precipitation, PM_2.5_, PM_10_, CO_2_, global irradiance, temperature, barometric pressure, and wind speed.	MWSN–IoT with an Edge–Fog–Cloud architecture, consisting of a Mobile Sensor Node (Edge) and a fixed Sink Node (Fog). The sensor node integrates low-cost sensors, GPS, Raspberry Pi, and XBee communication to connect to the Sink Node. The Sink Node, based on Raspberry Pi, communicates with the Cloud via Ethernet or Wi-Fi; it publishes the measurements to an MQTT broker and stores them in the MongoDB database in the Cloud using PyMongo 3.10.1. The Cloud integrates an MQTT broker, database, application server, and web application for real-time and historical monitoring.	At the Edge, measurements are spatially grouped, smoothed using means and medians, georeferenced, and corrected using calibration equations obtained against reference instruments. Frames are locally stored until successful delivery to the Fog is confirmed, enabling retransmission during communication failures. At the Fog, data integrity and duplicates are verified, validated measurements are published via MQTT and stored in MongoDB in the Cloud, with local temporary storage during service failures. In the Cloud, data are available for real-time visualization via WebSockets, historical retrieval, and georeferenced visualization using a 200 m spatial filter.	Prototypes of the Mobile Sensor Node and Sink Node are developed. An iterative and incremental methodology for the integrated development of prototype hardware and software is presented. The Cloud platform is configured, and the client application software is developed.	Experimental validation is performed on a mobile platform in a real urban environment to demonstrate a Technology Readiness Level (TRL) of 6. Sensor measurements are compared with those obtained from fixed reference stations.

**Table 2:** Environmental sensing subsystem hardware components.

Sensor	Function	Interface	Key Specifications
MPL115A2 (NXP Semiconductors N.V., Eindhoven, The Netherlands)	Barometric pressure and temperature	I^2^C	Pressure range: 50–115 kPa; pressure resolution: 0.15 kPa; accuracy: ±1 kPa; conversion time: 1.6 ms typ., 3 ms max
Mobile Electronic Rain Gauge *	Rainfall	I^2^C (ADS1115, 16-bit ADC)	Three-level precipitation scale: 1. Dry (no rain): For values lt; 2.0 mm/s. 2. Light or moderate rain: For values ≥ 2.0 mm/s and lt; 15. mm/s. 3. Heavy rain or thunderstorms: For values ≥ 15 mm/s. The sensor operates with a verified relative error of 8.5% in the flow rate calculation
Atlas Scientific EZO-CO_2_ Kit (Atlas Scientific LLC, Long Island City, NY, USA) **	CO_2_ concentration	I^2^C	Range: 0–10,000 ppm; resolution: 1 ppm; accuracy: ±50 ppm ±5% of reading; response time: <90 s
Rika RK100-02 (Hunan Rika Electronic Tech Co., Ltd., Changsha, China) ***	Wind speed	I^2^C (ADS1115, 16-bit ADC)	Range: 0–45 m/s; resolution: 0.1 m/s; accuracy: ±(0.3 + 0.03 V) m/s; starting threshold: <0.5 m/s
Rika RK200-04 (Hunan Rika Electronic Tech Co., Ltd., Changsha, China)	Global solar radiation	I^2^C (ADS1115, 16-bit ADC)	Range: 0–1500 W/m^2^; resolution: 1 W/m^2^; spectral range: 300–1100 nm; response time: ≤500 ns (95%)
Honeywell HPMA115S0 (Honeywell International Inc., Charlotte, NC, USA)	Particulate matter concentration (PM_2.5_/PM_10_)	USB (UART–USB)	Range: 0–1000 μg/m^3^; resolution: 1 μg/m^3^; accuracy: ±15%; response time: <6 s

* Previously developed by the authors and published in [44]. The design and experimental validation of the rain gauge are presented in that work. ** The kit consists of the Atlas Scientific EZO-CO_2_ circuit and its corresponding measurement probe. The probe was installed inside a 3/4-inch PVC tee fitting, preventing direct exposure to solar radiation while providing mechanical protection and maintaining natural air circulation around the sensor. *** The anemometer measurements were compensated using the vehicle speed obtained from the GNSS receiver. The compensation assumes that the dominant wind component is aligned with the vehicle trajectory, which is a reasonable approximation for mobile measurements at moderate to high speeds. Under this assumption, scalar compensation was applied without decomposing the wind vector into directional components. The system does not incorporate either a wind direction sensor or a compass. Therefore, the compensation does not explicitly account for crosswind components or changes in vehicle orientation, which may introduce errors in wind speed estimation when the airflow direction differs significantly from the vehicle trajectory. This condition constitutes a limitation of the implemented compensation method.

**Table 3:** Components of the Monitoring Center Architecture.

Component	Technology/Protocol	Main Function
MQTT Broker (Eclipse Mosquitto)	MQTT	Messaging: receives environmental measurements published by sink nodes and distributes them to subscribed monitoring clients.
Database Server (MongoDB)	MongoDB Wire Protocol via PyMongo	Storage: provides persistent storage of environmental measurements and supports historical data retrieval.
Application Server (Node.js)	HTTP/REST	Logic: implements the REST API, processes client requests, retrieves historical measurements from MongoDB, and returns responses in JSON format.
Web Application (HTML5, CSS, JavaScript, Leaflet + OpenStreetMap)	MQTT over WebSockets	Real-time monitoring: acts as an MQTT client, dynamically subscribing to environmental variables and displaying real-time measurements and system status.
	HTTP/REST	Historical monitoring: retrieves historical measurements from the application server, supports data queries and downloads, and visualizes environmental information.
	HTTP	Map visualization service: provides interactive visualization of georeferenced environmental measurements on digital maps.

**Table 4:** Technology Readiness Level (TRL) assessment of the proposed system.

TRL Assessment Category		Mobile Sensor Node (Edge Device	Sink Node (Fog Node)	Monitoring Center (Cloud Platform)
**Demonstration Units**	Concept			
	Breadboard			
	Brassboard			
	Developmental Model			
	Prototype	X	X	X
	Flight Qualified (Adapted to Pre-production prototype)			
**Environment**	Laboratory Environment	X	X	X
	Relevant Environment	X	X	X
	Space Environment (Adapted to Operational Urban Environment			
	Space Launch Operation/(Adapted to City-Wide Fleet Operation			
**Unit Description**	Form	X	X	X
	Fit	X	X	X
	Function	X	X	X
	Appropriate Scale	X		
**Overall TRL**		6	6	6

**Table 5:** Calibration Parameters.

Variable	RMSE_raw_	MNB_raw_	Slope	Intercept	R^2^
Temperature (MPL115A2)	3.40 °C	7.32%	0.4661	14.2070	0.872
Barometric Pressure (MPL115A2)	1.448 hPa	0.135%	0.967	32.20	0.717
Wind Speed (RK100-02)	0.289 m/s	−6.422%	0.761	0.7166	0.945
Global Irradiance (RK200-04)	82.585 W/m^2^	−11.51% *	1.149	16.624	0.993
PM_2.5_ (HPMA115S0)	36.248 µg/m^3^	575.809%	0.3486	−6.012	0.719
PM_10_ (HPMA115S0)	14.311 µg/m^3^	104.06%	0.7563	2.2219	0.686

* Low solar irradiance values (<50 W/m^2^) were excluded from the Mean Normalized Bias (MNB) calculation because of the sensor’s limitations.

**Table 6:** Post-calibration performance by environmental variable.

Variable	RMSE_cal_	MNB_cal_	NRMSE
Temperature (MPL115A2)	0.8112 °C	0.0729%	11.947%
Barometric Pressure (MPL115A2)	0.499 hPa	0.00002%	14.972%
Wind Speed (RK100-02)	0.164 m/s	0.573%	6.1527%
Global Irradiance (RK200-04)	31.087 W/m^2^	−2.85% *	2.887%
PM_2.5_ (HPMA115S0)	3.596 µg/m^3^	−11.9858%	18.404%
PM_10_ (HPMA115S0)	9.301 µg/m^3^	78.093%	13.551%

* For Global Irradiance, measurements below 50 W/m^2^ were excluded from the Mean Normalized Bias (MNB) calculation because of the sensor’s limitations.

**Table 7:** Performance before and after calibration using the independent validation dataset.

Variable	RMSE_raw_	MNB_raw_	NRMSE_raw_	RMSE_cal_	MNB_cal_	NRMSE_cal_
Temperature (MPL115A2)	0.400 °C	0.4384%	6.415%	1.667 °C	−3.903%	26.721%
Barometric Pressure (MPL115A2)	1.575 hPa	0.085%	42.453%	1.481 hPa	−0.053%	39.92%
Wind Speed (RK100-02)	1.038 m/s	−18.006%	36.678%	1.250 m/s	−23.251%	44.179%
Global Irradiance (RK200-04)	75.466 W/m^2^	−8.375% *	11.178%	61.082 W/m^2^	1.7839% *	9.048%
PM_2.5_ (HPMA115S0)	54.367 µg/m^3^	211.487%	264.431%	8.284 µg/m^3^	−14.333%	40.292%
PM_10_ (HPMA115S0)	12.561 µg/m^3^	14.983%	34.805%	11.416 µg/m^3^	−10.163%	31.638%

* For Global Irradiance, measurements below 50 W/m^2^ were excluded from the Mean Normalized Bias (MNB) calculation because of the sensor’s limitations.

## Data Availability

The raw data supporting the conclusions of this article will be made available by the authors on request.
